# A biomarker-guided framework for corticosteroid treatment stratification in acute exacerbation of idiopathic pulmonary fibrosis

**DOI:** 10.3389/fphar.2026.1934548

**Published:** 2026-08-24

**Authors:** Qinglong Chen, Yingying Zhang, Jialu Li, Qingjun Zhu, Ziyue Wang, Xin Wen

**Affiliations:** 1 College of Traditional Chinese Medicine, Shandong University of Traditional Chinese Medicine, Jinan, China; 2 College of Medicine, Shandong University of Traditional Chinese Medicine, Jinan, China; 3 Key Laboratory of Traditional Chinese Medicine Classical Theory, Ministry of Education, Shandong University of Traditional Chinese Medicine, Jinan, China; 4 Shandong Key Laboratory of Innovation and Application Research in Basic Theory of Traditional Chinese Medicine, Shandong University of Traditional Chinese Medicine, Jinan, China

**Keywords:** acute exacerbation, biomarkers, corticosteroids, idiopathic pulmonary fibrosis, precision pharmacotherapy, treatment stratification

## Abstract

Acute exacerbation of idiopathic pulmonary fibrosis is a fatal acute-on-chronic respiratory event with limited evidence-based treatment options. Systemic corticosteroids remain widely used, yet their benefit in unselected patients is uncertain, and indiscriminate immunosuppression may expose some patients to harm. A major translational gap is that most available biomarkers describe diagnosis, risk, or prognosis, but do not identify whether corticosteroids actually modify patient outcomes. This Hypothesis and Theory article proposes a testable, biomarker-guided conceptual framework that reframes corticosteroid treatment in acute exacerbation of idiopathic pulmonary fibrosis—from the question of average benefit in unselected patients to the hypothesis that corticosteroid effects differ across corticosteroid-responsive, corticosteroid-neutral, and corticosteroid-harm-prone biological states. We synthesise evidence on disease biology, corticosteroid outcome data, and candidate circulating, imaging, microbiological, and physiological biomarkers. Epithelial injury, inflammatory activation, fibroproliferation, endothelial and coagulation injury, infection or other trigger burden, radiological extent, oxygenation, and early clinical trajectory are evaluated as components of a treatment-stratification framework. We emphasise that these markers currently function mainly as diagnostic, risk, prognostic, safety, or dynamic-response markers rather than validated predictors of corticosteroid response. To bridge this gap, we propose a translational framework that integrates baseline biological signals with structured reassessment at 72–96 h. This framework separates three decision domains: inflammatory reversibility (the likelihood of corticosteroid-modifiable sterile inflammation), irreversible epithelial/fibroproliferative injury (the burden of structural damage unlikely to be modified by corticosteroids), and infection- or host vulnerability-related harm risk (the probability that immunosuppression is unsafe). It is intended to guide prospective treatment-aware cohorts, biomarker-by-treatment interaction analyses, target-trial emulation, and biomarker-enriched or adaptive trials.

## 1 Introduction

Idiopathic pulmonary fibrosis (IPF) is a chronic, progressive fibrosing interstitial pneumonia of unknown cause, characterised histologically and radiologically by a usual interstitial pneumonia (UIP) pattern and occurring predominantly in older adults (Raghu et al., 2022). Despite the introduction of the antifibrotic agents nintedanib and pirfenidone, IPF retains a dismal prognosis, with a median survival of only 2–3 years from diagnosis (King et al., 2011; Raghu et al., 2022). The clinical course, however, is heterogeneous and unpredictable: while many patients follow a course of gradual functional decline, a substantial proportion experience episodes of abrupt, clinically significant respiratory deterioration superimposed on their underlying disease (King et al., 2011). When such deterioration cannot be fully attributed to an identifiable cause such as cardiac failure or fluid overload, it is termed an acute exacerbation of IPF (AE-IPF) (Collard et al., 2016).

AE-IPF is among the most consequential events in the disease. With an annual incidence of approximately 5%–15% and an in-hospital mortality consistently exceeding 50%, it accounts for nearly half of all IPF-related deaths (King et al., 2011; Collard et al., 2016; Yoo et al., 2022; Wang et al., 2024). Both triggered and idiopathic exacerbations converge on diffuse alveolar damage (DAD) superimposed on UIP, aligning AE-IPF mechanistically with acute lung injury (Ryerson et al., 2015; Collard et al., 2016).

Management remains a profound therapeutic challenge. No intervention has improved survival in adequately powered randomised trials, and supportive care remains the foundation of treatment (Collard et al., 2016; Srivali et al., 2025; Mondoni et al., 2026). In this vacuum, high-dose systemic corticosteroids have become the *de facto* global standard of care, administered to most patients despite a weak, low-quality guideline recommendation and repeated signals of harm from intensified immunosuppression (King et al., 2011; Farrand et al., 2020).

Recent systematic reviews and large observational studies have found no consistent survival benefit from corticosteroids in AE-IPF, with signals of possible harm (Koshy et al., 2024; Mari et al., 2025). Yet a uniformly applied anti-inflammatory therapy would be expected to help some patients while being inert or harmful in others, because exacerbations engage distinct pathways of epithelial injury, innate inflammation, vascular dysfunction, and dysregulated repair (Chen et al., 2024). These developments motivate a shift in the central question from whether corticosteroids work in AE-IPF to in whom they might work—a shift that requires biomarkers capable of distinguishing treatment-responsive from treatment-unresponsive patients. Almost all available markers, however, are diagnostic or prognostic; a validated predictive biomarker of corticosteroid effect is conspicuously absent (Maher et al., 2017; Zinellu et al., 2023).

In this Hypothesis and Theory article, we synthesise current understanding of the pathobiology of AE-IPF, critically appraise the conflicting evidence on corticosteroid efficacy and harm, and survey the biomarker landscape with explicit attention to the distinction between prognostic and predictive markers. Drawing on lessons from stratified medicine in ARDS and other heterogeneous syndromes, we evaluate the feasibility of biomarker-guided treatment stratification and propose a research roadmap towards identifying the patients with AE-IPF who stand to benefit from corticosteroids.

## 2 Conceptual and pathobiological background

### 2.1 Evolution of the definition of AE-IPF and its consequences for treatment

The way AE-IPF is defined determines which biological events are grouped under a single label, and therefore how its treatment should be conceived. The 2007 Idiopathic Pulmonary Fibrosis Clinical Research Network criteria required the deterioration to be of unidentifiable cause, framing AE-IPF as an intrinsic acceleration of fibrosis and implicitly encouraging a uniform immunosuppressive response (Collard et al., 2007). The 2016 international working group removed this requirement: exacerbations are now classified as triggered or idiopathic, unified by a shared final common pathway of DAD superimposed on UIP rather than by a shared aetiology (Collard et al., 2016).

This shift carries an under-appreciated consequence for the corticosteroid question. By design, the revised definition assembles a biologically heterogeneous population under one diagnostic umbrella, and autopsy studies confirm that the alveolar injury underlying these events is itself pathologically variable rather than uniform (Oda et al., 2014). Yet the constituent subgroups may respond to corticosteroids in opposing directions. An idiopathic exacerbation driven by sterile inflammation might plausibly benefit from immunosuppression, whereas an infection-triggered event with active microbial replication may instead be worsened by it, since suppressing host defence can promote pathogen proliferation; an aspiration-related injury falls somewhere between the two. All three are nonetheless eligible for the same diagnosis and, in current practice, receive the same empirical corticosteroid therapy. This concern is not merely theoretical. In a national cohort of patients with rapidly progressive IPF, high-dose intravenous cyclophosphamide added to corticosteroids was independently associated with increased mortality, while co-trimoxazole and macrolides were associated with improved survival, indicating that agents directed at different biological targets produce divergent outcomes within an apparently single syndrome (Oda et al., 2016). A single averaged estimate of corticosteroid effect across all-comers is therefore unlikely to be clinically meaningful.

### 2.2 Diffuse alveolar damage on UIP: a shared morphology, not a shared therapy

The histopathological hallmark of AE-IPF is DAD superimposed on UIP, a temporally evolving pattern that progresses from early exudative injury to organising and fibroproliferative phases (Podolanczuk et al., 2023). The same broad morphology underlies ARDS, an analogy that has provided useful mechanistic insight.

Two caveats temper any direct inference from shared morphology to shared therapy. First, DAD is a final common pathway reachable from multiple upstream triggers, so identical histology does not imply identical active biology when treatment is being considered (Chen et al., 2024; Luo and Xiang, 2024). Second, AE-IPF arises in a chronically fibrotic lung with limited recruitable reserve, and corticosteroid effects in ARDS cannot be extrapolated to AE-IPF (Qadir et al., 2024). Because biopsy is rarely safe in critically hypoxaemic patients, the underlying biology must be inferred indirectly (Drakopanagiotakis et al., 2023), and biomarkers are needed to bridge this inferential gap.

### 2.3 The major biological and response axes of AE-IPF

If AE-IPF is best understood not as a single entity but as a convergence of pathways on a common morphological endpoint, then a useful organising principle is to map the principal biological axes that contribute, in varying proportions, to each event. We use five primary biological axes plus a cross-cutting imaging, physiology, and temporal-dynamics layer as a pragmatic working taxonomy of the measurable biology of AE-IPF rather than as validated endotypes. Each axis is associated with candidate molecular, circulating, microbiological, radiological, or physiological read-outs, and each carries distinct implications for the plausibility of corticosteroid benefit, neutrality, or harm (Supplementary Table S1).

These axes are not mutually exclusive, and the framework seeks to identify the dominant pattern rather than assign rigid endotypes. In broad terms, inflammatory activation nominates a candidate benefit signal, infection or another active trigger defines a candidate harm boundary, epithelial, fibroproliferative, and endothelial–coagulation injury indicate structural burden or limited reversibility, and serial imaging and physiology capture treatment-associated trajectory. These roles remain hypothesis-generating; the supporting evidence is examined in Section 4.

### 2.4 A biomarker taxonomy: the conceptual backbone of stratification

The AE-IPF biomarker literature has often conflated distinct evidentiary roles. We therefore adopt a six-category taxonomy that specifies what a biomarker can and cannot justify in a corticosteroid-stratification framework; the dominant category for each biological axis is summarised in Supplementary Table S1.

**FIGURE 1:**
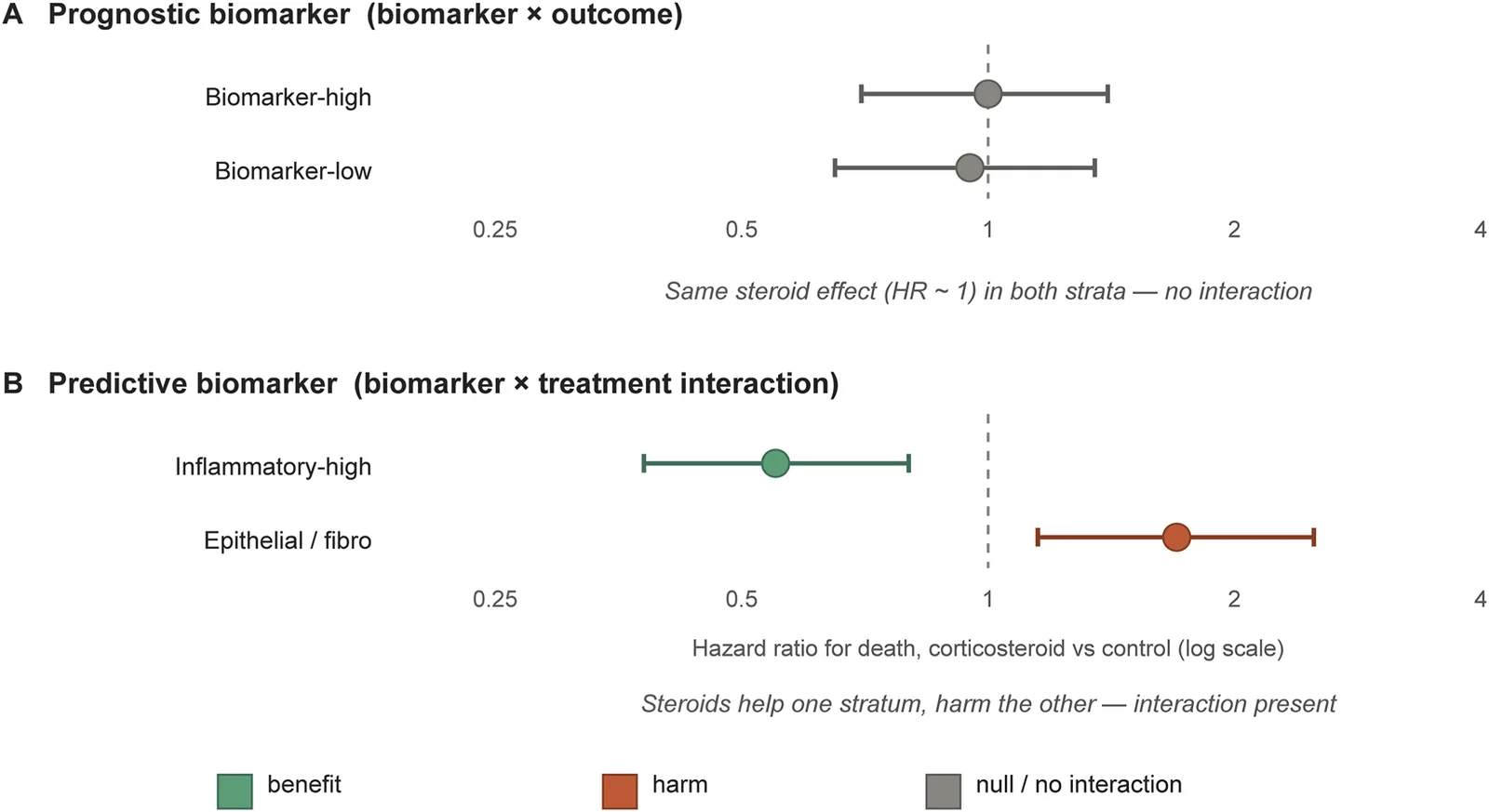
Limitations of prognostic biomarkers in guiding corticosteroid use. The figure illustrates hypothetical corticosteroid treatment effects within biomarker-defined strata, expressed as hazard ratios (HRs) for death for corticosteroid treatment *versus* control on a logarithmic scale. **(A)** Prognostic biomarker: biomarker-high and biomarker-low strata may differ in baseline risk, but the corticosteroid treatment effect remains similar in both groups, with HRs close to 1 and no biomarker-by-treatment interaction. Such a marker identifies patients at increased risk but does not identify those who are more likely to benefit from corticosteroids. **(B)** Predictive biomarker: corticosteroids are associated with a potentially favourable effect in an inflammation-dominant stratum and a potentially harmful effect in an epithelial injury- or fibroproliferation-dominant stratum, indicating a biomarker-by-treatment interaction and differential treatment response. HR <1 favours corticosteroids, HR >1 favours control, and HR ≈ 1 indicates a null effect or no interaction. All point estimates and confidence intervals are illustrative. No biomarker has yet been validated as predictive of corticosteroid response in acute exacerbation of idiopathic pulmonary fibrosis; demonstrating such an interaction is a central objective of this review.

Diagnostic biomarkers help establish AE-IPF and exclude mimics such as heart failure, pulmonary embolism, infection, or drug-induced lung injury (Collard et al., 2016). Risk biomarkers are measured during stable disease and predict future exacerbation (Zhang et al., 2021). Prognostic biomarkers are measured at or after exacerbation and predict outcomes irrespective of treatment; examples include oxygenation indices, LDH, CRP, KL-6, NLR, and diffuse HRCT involvement (Sakamoto et al., 2022). Predictive biomarkers, by contrast, identify biomarker-defined strata in which the effect of a specific therapy differs. Safety or harm biomarkers identify contexts in which the expected toxicity of corticosteroids may outweigh benefit, such as active infection or pre-existing immunosuppression (Idiopathic Pulmonary Fibrosis Clinical Research Network et al., 2012). Dynamic-response biomarkers capture early post-treatment trajectories, including changes in oxygenation, oxygen requirement, inflammatory markers, LDH, or radiological progression (Anan et al., 2022). A prognostic association alone is therefore insufficient for treatment selection: a biomarker becomes predictive only through a demonstrated biomarker-by-corticosteroid interaction, a standard no AE-IPF biomarker has yet met (Calfee et al., 2014; 2018; Hernán et al., 2016; Enomoto et al., 2018) (Figure 1).

## 3 Current evidence on corticosteroid therapy in AE-IPF

### 3.1 A weak recommendation sustaining a near-universal practice

The clinical use of corticosteroids in AE-IPF is characterised by a striking mismatch between the weakness of the evidence supporting treatment and the consistency with which it is applied in practice. The 2011 ATS/ERS/JRS/ALAT guideline recommended corticosteroids for most patients with AE-IPF only as a weak recommendation based on very low-quality evidence, and explicitly stated that no specific recommendation could be made regarding dose, route, or duration (Raghu et al., 2011). Subsequent expert commentary has reiterated that this recommendation rests more on the high mortality of AE-IPF and the absence of established alternatives than on controlled evidence of efficacy (Hung and Raghu, 2024). Nevertheless, corticosteroids remain deeply embedded in routine care. In an international survey of 509 pulmonologists with expertise in interstitial lung disease from 66 countries, high-dose corticosteroids were administered by 94% of respondents, while only 4% reported never using immunosuppression, underscoring the extent to which a weakly supported intervention has become a near-universal practice (Kreuter et al., 2020). Approaches to prevention, diagnosis, and treatment also varied substantially across regions, indicating that current practice reflects therapeutic uncertainty rather than a settled standard of care. This uncertainty has direct methodological consequences: when corticosteroid use and dose are determined by clinician judgement rather than protocol, patients receiving more intensive regimens are often those with greater respiratory severity, thereby embedding confounding by indication into observational comparisons from the outset.

### 3.2 An uncertain and possibly adverse average effect

The accumulated outcome evidence does not demonstrate a survival benefit from corticosteroids in unselected AE-IPF, and recent syntheses increasingly suggest that any average effect is uncertain and may be unfavourable. In a propensity-weighted observational cohort, corticosteroid treatment was not associated with reduced in-hospital mortality, while treated patients were more likely to require intensive care and mechanical ventilation, illustrating the severity imbalance that complicates causal interpretation (Farrand et al., 2020). A 2025 systematic review of acute exacerbations of interstitial lung disease further sharpened this distinction: corticosteroids were associated with improved outcomes in non-IPF ILD exacerbations, but the same benefit was not demonstrated in AE-IPF, suggesting that the fibrotic IPF substrate may fundamentally alter treatment responsiveness (Srivali et al., 2025). A separate 2025 meta-analysis focused on AE-IPF reported an association between corticosteroid exposure and increased mortality, although the authors acknowledged the observational nature and heterogeneity of the included studies (Mari et al., 2025). Most recently, a 2026 Respiratory Research systematic review and meta-analysis found persistently high mortality among corticosteroid-treated AE-IPF patients, with pooled 90-day and in-hospital mortality of approximately 42% and 43%, respectively (Mondoni et al., 2026). These data should not be interpreted as definitive proof that corticosteroids are intrinsically harmful, because more aggressive regimens are often reserved for the sickest patients. Rather, they show that the average corticosteroid effect in AE-IPF remains inseparable from confounding by indication, treatment heterogeneity, and biological heterogeneity. More unstratified observational studies are therefore unlikely to resolve the question; they will mainly re-estimate a contaminated average effect.

### 3.3 Clinical signals against indiscriminate immunosuppression

Two further lines of evidence are pivotal, because they begin to expose the heterogeneity that an averaged estimate conceals. The first is that the apparent effect of corticosteroids is not uniform across acute exacerbations of fibrotic lung disease. High-dose corticosteroids are associated with improved outcomes in acute exacerbations of non-IPF interstitial lung disease, in which an inflammatory component is generally more prominent, whereas the same benefit is not demonstrable in AE-IPF (Srivali et al., 2025). This contrast indicates that responsiveness tracks with the underlying biology rather than with the morphology of the acute syndrome, so that “acute exacerbation” is too coarse a category to predict treatment response. The second is that intensifying immunosuppression has been tested directly and found harmful. In the randomised EXAFIP trial, adding cyclophosphamide to glucocorticoids increased 3-month mortality rather than reducing it (Naccache et al., 2022). This finding echoes the established harm of combination immunosuppression in stable IPF, where the same intuition that more suppression should help proved fatal (Idiopathic Pulmonary Fibrosis Clinical Research Network et al., 2012). Together these results argue that escalating immunosuppression is not a rational default in AE-IPF, and that the central problem is one of patient selection rather than of treatment intensity. We do not attempt an exhaustive account of dose, timing, and tapering here, noting only that the optimal regimen remains undefined and that early dose tapering has been associated with lower in-hospital mortality (Anan et al., 2022).

### 3.4 Reframing the clinical question

These observations describe a genuine clinical dilemma rather than a simple shortage of data. The natural response is to call for a larger, definitive randomised trial of corticosteroids *versus* placebo, and such a trial is both needed and underway. A trial that estimates only an overall average effect, however, risks reproducing the very problem it seeks to solve, because an average treatment effect can conceal variation across patients and wrongly suggest that an intervention is inert when it in fact benefits some and harms others (Graham Linck et al., 2024). If AE-IPF comprises subgroups in which corticosteroids help, do nothing, or cause harm, averaging across them will again yield a diluted, near-null estimate that describes no individual patient. The limitation of the current evidence is therefore not principally its quantity but its framing. Whether corticosteroids should be used in AE-IPF is a question that biological heterogeneity has rendered nearly unanswerable. The answerable and clinically actionable question is in whom they should be used: which patients occupy a corticosteroid-responsive biological state, which are likely to derive no benefit, and which stand to be harmed. Answering this question requires markers capable of distinguishing these states, to which we now turn.

## 4 Candidate biomarkers for stratifying corticosteroid response

### 4.1 From individual biomarkers to corticosteroid-relevant biological signals

Having reframed the clinical question as one of patient selection, we now ask which measurable signals might distinguish the candidate states. Almost no AE-IPF biomarker is predictive in the formal sense, and even in large contemporary IPF cohorts the leading circulating markers have remained prognostic for progression and mortality rather than demonstrated modifiers of treatment effect (Randall et al., 2026). The markers discussed below are therefore nominated by prognostic association and biological reasoning rather than by any proven interaction with corticosteroid response. We consider them not as a catalogue but by the biological axis they reflect and the corticosteroid-relevant question each may help answer, namely, whether it raises the prior plausibility of benefit, neutrality, or harm (Supplementary Table S1). For every axis, the proposed role is a hypothesis to be tested, not a conclusion to be applied. A note on organisation is warranted. Sections 4.2–4.6 survey circulating and microbiological markers according to the five primary pathobiological axes defined in Section 2.3 and Supplementary Table S1. Sections 4.7 and 4.8 are instead organised by measurement modality, because imaging and physiological read-outs are cross-cutting: they integrate signals from several axes simultaneously and, in the case of serial physiology, capture the dynamic response to treatment rather than any single biological process. Section 4.9 then integrates all domains into a candidate panel. Each marker is therefore placed where it contributes the most decision-relevant information.

### 4.2 Alveolar epithelial injury markers: markers of damage, not necessarily of steroid responsiveness

Alveolar epithelial injury is central both to stable IPF progression and to the acute event, in which diffuse alveolar damage superimposed on usual interstitial pneumonia entails injury to type II pneumocytes, disruption of the alveolar epithelial barrier, and failed repair. Circulating markers of this axis include Krebs von den Lungen-6 (KL-6), surfactant proteins A and D (SP-A, SP-D), growth differentiation factor-15 (GDF-15), the soluble receptor for advanced glycation end-products (sRAGE), human epididymis protein 4 (HE4/WFDC2), and, less specifically, lactate dehydrogenase (LDH). These markers are attractive because they are mechanistically tied to the injured epithelial surface, are measurable in blood, and are reproducibly associated with disease severity, exacerbation risk, or mortality (Maher et al., 2017; Zinellu et al., 2023).

KL-6, a mucin-like glycoprotein shed by injured and regenerating type II pneumocytes, is the most extensively studied. It is elevated in fibrotic interstitial lung disease, rises further at exacerbation, and is associated with worse survival, and a systematic review and meta-analysis confirms that elevated KL-6 predicts severity, progression, acute exacerbation, and poor outcome across interstitial lung disease (Zhang et al., 2021). Baseline KL-6 also predicts the future occurrence of an exacerbation, with a threshold near 1300 U/mL identified in prospective data, whereas CCL18 measured in the same cohort showed no such predictive value, an early illustration that biological candidacy and validated clinical utility are not the same (Ohshimo et al., 2014). More recent longitudinal work reinforces this trajectory dependence, since serial KL-6 measurements and their rate of change discriminate patients who go on to exacerbate from those who remain stable, and a rising LDH is independently associated with mortality in the same analyses (Choe et al., 2025). SP-D and SP-A behave similarly, reflecting epithelial damage and barrier permeability, and a pooled analysis of more than three thousand five hundred patients found that higher serum SP-D tracks ILD occurrence, progression, acute exacerbation, and death (He et al., 2025). GDF-15, a stress-responsive member of the TGF-beta superfamily, is markedly elevated in AE-IPF and independently predicts mortality above approximately 1,075 pg/mL (Cao et al., 2022), and HE4/WFDC2, localised to bronchiolar and injured epithelium, tracks disease severity and has also been incorporated into recent IPF survival models (Tian et al., 2024).

Several recent studies describe epithelial-axis or related innate immune signalling markers as predictive biomarkers for acute exacerbation, including serum sRAGE in a combined German and Japanese cohort and soluble Toll-like receptor 4 in a separate Japanese cohort, yet in each case the term denotes prediction of the future event rather than modification of a treatment effect (Kitadai et al., 2025). Similarly, serum metabolomic profiling has identified 5-HETE as a candidate marker for AE-IPF diagnosis and short-term prognosis, but this evidence also predicts event status or outcome rather than corticosteroid treatment effect (Zhao et al., 2025). In the terminology adopted in Section 2.4 these remain risk or prognostic markers, because none was evaluated for an interaction with corticosteroid therapy. A useful contrast is provided by epithelial markers tested in randomised antifibrotic trials rather than in corticosteroid-treated AE-IPF cohorts. In a *post hoc* analysis of a randomised placebo-controlled phase 3 trial, baseline SP-D identified the subgroup of patients with IPF in whom pirfenidone, rather than corticosteroids, slowed functional decline, demonstrating that a treatment-effect-modifying signal can in principle be extracted from this axis, but only against a defined intervention in a randomised design (Ikeda et al., 2020). No comparable analysis exists for corticosteroids in AE-IPF.

For corticosteroid stratification, the biology of this axis carries a counter-intuitive implication that must be made explicit, because reading it backwards would be clinically harmful. A high epithelial-injury signal plausibly marks an extensive and partly irreversible burden of acute structural damage rather than a reversible inflammatory state, a reading consistent with autopsy data showing that the alveolar injury underlying these events is pathologically heterogeneous rather than uniformly inflammatory (Oda et al., 2014). In such patients corticosteroids might suppress inflammatory bystanders without altering the dominant pathological trajectory. High KL-6, GDF-15, HE4, or LDH should therefore be treated as a candidate component of a corticosteroid-neutral signature, especially when accompanied by severe oxygenation failure and extensive HRCT abnormality, not as a marker of responsiveness, and certainly not as an indication to intensify immunosuppression, which would invert its likely meaning. Crucially, every association cited here is prognostic and was established in cohorts in which most or all patients received corticosteroids, and none has been tested for interaction with treatment. The clinically useful question for future study is therefore not whether a high epithelial-injury signature predicts death, but whether, among patients receiving comparable supportive care, it identifies those in whom corticosteroids confer no benefit despite high baseline risk.

### 4.3 Fibroproliferation and matrix-remodelling markers: identifying a structurally dominant phenotype

A closely related axis reflects fibroproliferation and extracellular-matrix remodelling, and it matters because the acute event always arises on a background of established UIP in which fibroblast activation and architectural distortion are already advanced. Matrix metalloproteinase-7 (MMP-7) is among the most reproducible prognostic markers of progression and mortality in IPF, and together with CCL18 it was confirmed as a predictor of progression in the largest IPF biomarker cohort to date (Maher et al., 2017). Its prognostic weight has since been consolidated by an individual participant data meta-analysis, in which each standard deviation increase in baseline MMP-7 carried an approximately 23 per cent rise in mortality risk, and it was subsequently validated against an independent threshold in a separate cohort (Khan et al., 2022). The same signal recurs in contemporary randomised antifibrotic trials, where MMP-7 and CCL18 emerged among the strongest blood predictors of all-cause mortality alongside diffusing capacity (Randall et al., 2026). Osteopontin, a matricellular driver of fibroblast recruitment, is elevated in AE-IPF and carries prognostic value (Gui et al., 2020), and broader expression studies confirm that higher osteopontin marks poor outcome in IPF and feed it into composite progression indices that combine osteopontin, MMP-7, intercellular adhesion molecule-1, and periostin (Ji et al., 2023; Zhu et al., 2024). Periostin behaves similarly, with higher circulating levels independently associated with worse survival across prospective cohorts and a recent prognostic meta-analysis (Okamoto et al., 2023; Alghwyeen et al., 2025). Circulating fibrocytes, the mesenchymal progenitors of fibrosis, rise during exacerbation and were originally reported to independently predict early mortality above a 5 per cent threshold of total blood leucocytes (Moeller et al., 2009). HE4 and CCL18 straddle this axis and the epithelial and inflammatory ones.

The fibrocyte literature also illustrates why these associations must be handled cautiously, because the original 5 per cent threshold did not reproduce when tested in the prospective PROFILE cohort, where prognostic significance emerged only at an empirically derived cut point near 2.2 per cent and only for overall mortality rather than for lung function decline or disease activity (Stewart et al., 2021). A marker that appears robust in a derivation cohort can therefore weaken or shift substantially on external validation, a recurring caution for every candidate in this section.

The conceptual position for treatment is the same as for the epithelial axis, and equally hypothesis-generating. A phenotype dominated by fibroproliferative markers is predicted to be relatively corticosteroid-unresponsive, because corticosteroids act on inflammatory gene expression, cytokine production, leucocyte trafficking, and vascular permeability rather than on established matrix deposition, fibroblastic foci, or architectural distortion (Luo and Xiang, 2024). This does not mean such patients should never receive corticosteroids under current practice. It means their inclusion in unselected cohorts may dilute any benefit concentrated in a smaller inflammatory subgroup, one mechanistic explanation for the near-null average effect documented in Section 3. A practical corollary is that these markers should not be read in isolation, since the informative quantity may be not the absolute level of any single remodelling marker but the balance between fibroproliferative burden and inflammatory activity. A patient with high MMP-7, extensive background fibrosis, and minimal systemic inflammation occupies a different biological state, and a different expected corticosteroid response, from one with high inflammatory markers, new ground-glass opacity (GGO), and limited established fibrosis. Notably, the few biomarkers from this axis that have been examined for treatment-effect modification were tested against antifibrotic agents rather than corticosteroids. Higher baseline periostin, for instance, was associated with a more favourable response to nintedanib in a prospective cohort (Okamoto et al., 2023), which underscores that a treatment-interaction signal is in principle obtainable from this axis but has never been sought for corticosteroids. As with every axis, this balance has not been tested through a biomarker-by-treatment interaction analysis and must not be presented as if it had.

### 4.4 Inflammatory and immune-activation markers as plausible but unvalidated benefit markers

If any axis offers candidate benefit markers, markers that might positively identify responders, it is this one, because it represents the component of AE-IPF most likely to be modifiable by anti-inflammatory therapy. AE-IPF is not merely a linear acceleration of chronic fibrosis, since in at least a subset of patients acute inflammatory amplification, innate immune activation, neutrophil recruitment, and macrophage activation contribute to the clinical event (Chen et al., 2024; Koshy et al., 2024). Widely available markers include C-reactive protein (CRP), the neutrophil-to-lymphocyte ratio (NLR), white-cell and neutrophil counts, ferritin, and LDH, all associated with outcome in AE-IPF (Collard et al., 2007). The NLR illustrates both the appeal and the limits of this group, since a higher value at presentation independently predicts 90-day mortality in AE-IPF and an early rise tracks oxygenation deterioration, yet it remains a nonspecific index of the innate to adaptive balance rather than a measure of corticosteroid-modifiable inflammation (Arai et al., 2023; Monteleone et al., 2025). More mechanistic signals extend the picture. Interleukin-6 is elevated at exacerbation and independently predicts both the occurrence of acute exacerbation and subsequent mortality across interstitial lung disease, with interleukin-8 rising in parallel (Papiris et al., 2018; Lee et al., 2021). The neutrophil-derived calcium-binding proteins S100A8 and S100A9, together with high-mobility group box 1, rise at the onset of exacerbation relative to stable disease and independently predict 3-month mortality, with hazard ratios of approximately four (Yamaguchi et al., 2020; Tanaka et al., 2021; Kitadai et al., 2025). A panel of soluble mediators, including soluble urokinase plasminogen activator receptor, soluble intercellular adhesion molecule-1, macrophage migration inhibitory factor, and interleukin-1 beta, has been linked to short-term mortality (Li et al., 2020). Serum amyloid A (SAA), an acute-phase apolipoprotein induced downstream of IL-6 and IL-1 signalling, deserves mention within this axis. SAA rises in interstitial lung disease and has been associated with disease activity and short-term outcomes in AE-IPF, plausibly reflecting hepatic amplification of the same innate inflammatory signal captured by CRP, IL-6, and S100A8/A9. It was not included among the core candidates because current evidence derives from small single-centre cohorts, assay standardisation is limited, and, like CRP, SAA cannot separate sterile inflammation from infection. It is best regarded as a supplementary component of a composite inflammatory signature pending prospective, treatment-aware validation.

The theoretical appeal is clear. If corticosteroids benefit any subgroup, it is biologically most plausible that the benefit is concentrated among patients in whom sterile, predominantly non-infectious inflammation drives deterioration, presenting perhaps with abrupt worsening, markedly elevated CRP or NLR, raised IL-6 or IL-8, new GGO-predominant abnormality, and no strong microbiological evidence of active infection. This is the candidate corticosteroid-responsive phenotype that future studies should test (Section 5.2).

Yet inflammatory markers are also the easiest to over-interpret, and two cautions are decisive. First, an elevated CRP or neutrophilia does not distinguish sterile inflammation from infection, nor does it establish that inflammation is causal rather than secondary to severe epithelial injury, and greater severity is not the same as greater steroid responsiveness. A meta-analysis of prognostic factors in AE-IPF reinforces this, since a higher proportion of neutrophils in bronchoalveolar lavage during the event was independently associated with death, indicating that intense neutrophilic inflammation often marks worse disease rather than a more tractable one (Pitre et al., 2024). Second, and for this reason, the harm axis (Section 4.5) must be read simultaneously, because without it the very inflammatory signature that nominates a patient as a candidate responder may instead mark an infective trigger in whom immunosuppression is hazardous. These markers can become predictive only if shown to interact with treatment, that is if patients with a high inflammatory signature derive a greater corticosteroid effect than those with a low signature after accounting for infection and baseline severity. The most realistic instrument is likely a composite inflammatory signature combining CRP, NLR, IL-6 or IL-8 where available, HRCT pattern, and early oxygenation trajectory rather than any single marker, an approach already foreshadowed by simple multidimensional scores in which CRP, the PaO2 to FiO2 ratio, and a diffuse HRCT pattern jointly stratify 3-month mortality (Sakamoto et al., 2022).

### 4.5 Infection- and trigger-related markers: harm markers rather than benefit markers

The 2016 definition explicitly admits triggered exacerbations, including those associated with infection, aspiration, drug toxicity, and recent procedures, so that biologically distinct events are now grouped under one label (Section 2.1) (Collard et al., 2016). For stratification, infection- and trigger-related markers matter not because they predict benefit, but because they may identify patients in whom immunosuppression is dangerous. Procalcitonin is the most accessible candidate, while presepsin, microbiological culture, respiratory viral polymerase chain reaction, fungal markers, bronchoalveolar lavage, and metagenomic next-generation sequencing (mNGS) extend the workup, and lavage pepsin or bile acids may indicate aspiration where available. In acute exacerbation of fibrosing interstitial lung disease, lavage mNGS detects pathogens far more often than conventional culture and prompts adjustment of antimicrobial and immunosuppressive treatment in a meaningful proportion of patients, precisely the decision this axis is meant to inform (Zhan et al., 2024). Notably, a randomised AE-IPF study evaluated procalcitonin-guided antibiotic stewardship rather than treatment of the syndrome itself. A threshold of 0.25 ng/mL shortened antibiotic duration without changing mortality, supporting procalcitonin as a usable stewardship tool (Ding et al., 2013). This study should be distinguished from randomised therapeutic trials in AE-IPF, including thrombomodulin alfa and EXAFIP, as well as the ongoing EXAFIP2 glucocorticoid trial (Kondoh et al., 2020; Naccache et al., 2022; Fondation Hôpital Saint-Joseph, 2026).

The clinical logic of this axis is asymmetric. A negative infection workup does not prove corticosteroid benefit, but a positive infection signal substantially raises the probability of harm, because corticosteroids may impair pathogen clearance, promote secondary and fungal infection, and worsen outcomes when microbial replication is active. This is not merely theoretical, since a recent meta-analysis found pre-admission corticosteroid use independently associated with higher mortality in AE-IPF, consistent with net harm when immunosuppression is applied in the wrong biological context (Pitre et al., 2024). Infection markers should therefore enter corticosteroid decision-making as safety markers, and biomarker-stratified trials must separate infection-dominant from sterile inflammatory events, lest mixing the two obscure both benefit and harm.

Two difficulties bear directly on the benefit axis. First, distinguishing sterile inflammation from infection-triggered injury is genuinely hard, since fever, neutrophilia, and a raised CRP accompany the inflammatory axis of AE-IPF itself and are not specific for infection. Culture-independent studies show altered bacterial burden and composition during AE-IPF relative to stable disease, yet have not established that exacerbations represent acute bacterial infection rather than dysbiosis secondary to acute injury (Dickson et al., 2014; Molyneaux et al., 2017). Host susceptibility may further complicate this boundary: the TLR3 L412F variant has been associated with dysregulated lung microbiome composition, impaired antibacterial and antiviral fibroblast responses, and increased AE-related death in IPF (McElroy et al., 2022). Second, molecular detection of an organism does not establish causation, so colonisation, contamination, and low-level detection must be weighed against the clinical picture, a caveat that grows more pressing as mNGS sensitivity increases. The operative question is one of dominance, namely, whether infection is the dominant active driver at the moment corticosteroids are being considered, or a resolved or minor trigger in a patient now experiencing sterile inflammatory injury. A candidate corticosteroid-harm phenotype (Section 5.2) accordingly includes an elevated procalcitonin or presepsin, positive bacterial or viral testing, fungal markers, an immunosuppressed background, sepsis physiology, or multi-organ dysfunction, identifying patients who should be classified not merely as non-responders but as a distinct group in whom the risk to benefit balance is unfavourable.

### 4.6 Endothelial and coagulation markers: markers of severe alveolar–capillary injury

AE-IPF involves injury to the alveolar–capillary barrier and activation of coagulation alongside epithelial damage and immune activation. Markers in this domain, including thrombomodulin, D-dimer, von Willebrand factor, angiopoietins, protein C, and plasminogen activator inhibitor-1, reflect endothelial injury, microvascular dysfunction, and impaired fibrinolysis. This is consistent with the intermediate position of the AE-IPF plasma profile between stable disease and acute lung injury, in which epithelial, endothelial, coagulation, and inflammatory signals are simultaneously elevated (Collard et al., 2010). The biology is plausible, since tissue factor expressed by injured alveolar epithelium drives intra-alveolar fibrin deposition and thrombin generation, and angiopoietin-2 rises with endothelial permeability and tracks worse outcome in IPF, linking microvascular injury mechanistically to fibrogenesis (May et al., 2023).

For stratification these markers occupy an uncertain position. They may identify a severe, DAD-like phenotype with widespread alveolar–capillary injury and limited reversibility, or endothelial permeability injury may coexist with an inflammatory component that anti-inflammatory therapy could in principle modify, and current evidence cannot distinguish these possibilities. The negative randomised trial of thrombomodulin alfa is instructive, since targeting the coagulation axis alone did not improve outcomes in unselected AE-IPF (Kondoh et al., 2020), reinforcing the broader theme that a single-pathway intervention applied to all-comers is unlikely to succeed in a multi-axis syndrome. Endothelial and coagulation markers may yet contribute to a composite severity or endotype model, but they are not credible standalone predictors of corticosteroid benefit. Marked elevations should alert clinicians and investigators to severe alveolar–capillary injury and high baseline risk, but whether such patients should receive, avoid, or rapidly taper corticosteroids cannot be determined from this domain in isolation and requires integration with the inflammatory, infection, and dynamic-response axes.

### 4.7 Radiological markers: visible patterns of hidden biology

HRCT is central to the diagnosis of AE-IPF and may also inform stratification. Beyond establishing new bilateral GGO and/or consolidation on a UIP background, the distribution and extent of these abnormalities carry prognostic weight, and two distinct radiological dimensions must be kept separate. The first is distribution, in which Akira and colleagues classified new opacity as peripheral, multifocal, or diffuse, the diffuse pattern carrying a roughly four to five fold higher hazard of death than the peripheral pattern (Akira et al., 2008). The second is density, in which GGO-predominant abnormality may reflect potentially reversible exudative injury, whereas dense consolidation may indicate established organising injury or alveolar collapse. Conflating the two obscures the biology the image is meant to reveal.

The relevance to stratification is that HRCT may act as a macroscopic biomarker bridging the radiological and inflammatory axes. In the EXAFIP cohort a diffuse pattern was accompanied by markedly higher CRP than a peripheral pattern (Naccache et al., 2022), and composite scores combining oxygenation, CRP, and a diffuse pattern stratify 3-month mortality (Sakamoto et al., 2022). Yet a diffuse pattern is associated with both higher inflammatory markers and worse prognosis, and the latter may reflect advanced, poorly reversible injury rather than a steroid-responsive state, so HRCT is best regarded as a candidate component of a composite endotype rather than a standalone predictive marker.

Quantitative radiomics and machine-learning approaches measuring acute opacity extent, the GGO-to-consolidation ratio, and background fibrosis volume may outperform visual classification (Jacob et al., 2017). Deep-learning segmentation of baseline CT now predicts progression and mortality in IPF, and AI-derived measures of fibrotic burden and pulmonary vascular volume have consistently outperformed visual CT scores for prognostication, most promising when combined with CRP, KL-6, procalcitonin, and oxygenation indices (Thillai et al., 2024; Khalid et al., 2025). For future trials, baseline HRCT should be systematically scored or quantified rather than used only diagnostically, so that radiological phenotype can be tested for interaction with treatment effect.

### 4.8 Physiological and dynamic-response markers: the closest approximation to treatment response

Physiological markers are among the most actionable tools in AE-IPF because they can be measured serially and integrate the consequences of epithelial injury, inflammation, vascular dysfunction, and fibrotic reserve. Baseline PaO_2_/FiO_2_ and SpO_2_/FiO_2_ ratios, oxygen requirement, respiratory rate, the ROX index, and the need for high-flow nasal cannula or ventilatory support all carry prognostic information, yet, as throughout, severe baseline oxygenation failure marks high risk rather than steroid responsiveness.

The distinctive contribution of this axis is not prognostic but dynamic. Because AE-IPF evolves over days and no baseline marker predicts corticosteroid response, the short-term trajectory after treatment initiation may approximate the true treatment response more faithfully than any baseline value. Improvement in the SpO_2_/FiO_2_ or PaO_2_/FiO_2_ ratio over the first 72–96 h, a falling oxygen requirement, declining CRP, NLR, or LDH, and arrest of radiological progression are candidate dynamic-response markers, consistent with evidence that early corticosteroid tapering trajectory relates to outcome (Anan et al., 2022) and that corticosteroid responsiveness itself varies between AE-IPF and other ILD exacerbations (Jang et al., 2021). The reframing is clinically consequential, since a baseline marker asks whether corticosteroids should be started, whereas a dynamic-response marker asks whether they should be continued, tapered, or stopped. In a disease of high mortality and uncertain efficacy, the second question may be the more answerable. Early improvement may identify patients in whom corticosteroids are modifying a reversible component of the event, while persistent deterioration may justify early tapering, redirection to supportive care, transplant evaluation where appropriate, or avoidance of escalating immunosuppression.

Dynamic markers may also partly dissolve the predictive-biomarker problem. Even if no baseline marker reliably identifies responders, pre-specified day 3–4 reassessment rules based on SpO_2_/FiO_2_, CRP, LDH, and oxygen requirement could define response-adaptive strategies, testing not only whether corticosteroids should be given but whether early biological response can govern their duration. They are not, however, baseline predictive biomarkers and should not be presented as such.

### 4.9 Integrating candidate markers into a corticosteroid response panel

No single biomarker is likely to identify corticosteroid responders in AE-IPF, because the syndrome is biologically heterogeneous and each marker is non-specific in isolation. A more realistic strategy is to integrate markers into a multidimensional panel that conceptually organises three clinically relevant domains for future validation. These are inflammatory reversibility, irreversible epithelial/fibroproliferative injury, and infection- or host vulnerability-related harm risk. In this framework, CRP, NLR, IL-6, IL-8, S100A8/A9, and suPAR represent candidate inflammatory benefit signals. KL-6, SP-D, GDF-15, LDH, MMP-7, periostin, and fibrocyte-related markers index epithelial injury and remodelling burden. Procalcitonin, microbiological testing, viral and fungal markers, and clinical sepsis features define the safety boundary of immunosuppression. HRCT pattern, acute opacity extent, background fibrosis, and oxygenation trajectory then provide the radiological and dynamic context in which these signals should be interpreted.

The purpose of such a panel is not to generate another severity score, but to distinguish biological states with different expected corticosteroid responses. A marker that predicts death may identify high-risk patients without identifying steroid-responsive patients. A safety marker may be clinically useful even if it is not a strong mortality predictor, because it flags patients in whom immunosuppression may be harmful. A dynamic marker such as early improvement in SpO_2_/FiO_2_ may not predict baseline susceptibility, but it may help determine whether continued corticosteroid exposure remains rational. One further domain warrants brief consideration: constitutive host genetics. The MUC5B rs35705950 promoter variant, TOLLIP polymorphisms, and variants in telomere-maintenance genes such as TERT and TERC define biologically distinct IPF subsets involving mucociliary, immune, and ageing-related pathways. The TLR3 L412F variant further illustrates how genotype may influence antimicrobial responses and lung microbiome composition, with potential relevance to infection-associated exacerbation. Because germline variants are fixed, they cannot serve as dynamic-response markers but may inform baseline stratification. No variant has yet demonstrated differential corticosteroid responsiveness; targeted germline profiling should therefore be considered in future biomarker-guided precision cohorts.

## 5 A proposed biomarker-guided corticosteroid stratification framework

### 5.1 Core premise: from prognostic markers to testable decision hypotheses

The transition from prognostic biomarkers to treatment-predictive biomarkers should not be assumed. Combining diagnostic, prognostic, safety, and dynamic-response markers does not by itself create a validated predictive tool for corticosteroid response. Rather, the framework proposed here is a hypothesis-generating construct that organises biologically plausible but currently non-predictive signals into testable candidate response states. Its purpose is to define enrichment strata, stratification variables, and pre-specified biomarker-by-treatment interaction hypotheses for future treatment-aware cohorts, target-trial emulations, and biomarker-enriched or adaptive trials. In this sense, “corticosteroid-modifiable inflammation” should be read as a candidate biological state to be tested, not as a validated bedside indication for corticosteroid therapy. The biomarker panel outlined above should therefore not be treated as another severity score or as a current treatment-selection algorithm. Its purpose is to structure heterogeneous biological signals into three testable decision domains: the hypothesised likelihood of corticosteroid-modifiable sterile inflammation, the burden of irreversible epithelial–fibroproliferative injury, and the likelihood of corticosteroid-related harm. In this formulation, AE-IPF is positioned not within rigid diagnostic bins but on a dynamic benefit–harm plane, generating three provisional response states: corticosteroid-responsive, corticosteroid-neutral, and corticosteroid-harm-prone. This is deliberately a hypothesis-generating framework. No circulating, imaging, microbiological, or physiological marker has yet been validated as predictive of corticosteroid response in AE-IPF, and high baseline risk must not be mistaken for steroid responsiveness. The analogy with precision critical care is useful but limited: ARDS hyper- and hypo-inflammatory subphenotypes were empirically derived and then linked to differential treatment effects, with later work showing that parsimonious classifiers can approximate those states for trial use (Sinha et al., 2020). AE-IPF requires the same evidentiary sequence rather than rhetorical borrowing: derivation, validation, and then prospective testing of treatment interaction.

### 5.2 Candidate corticosteroid response states

**FIGURE 2:**
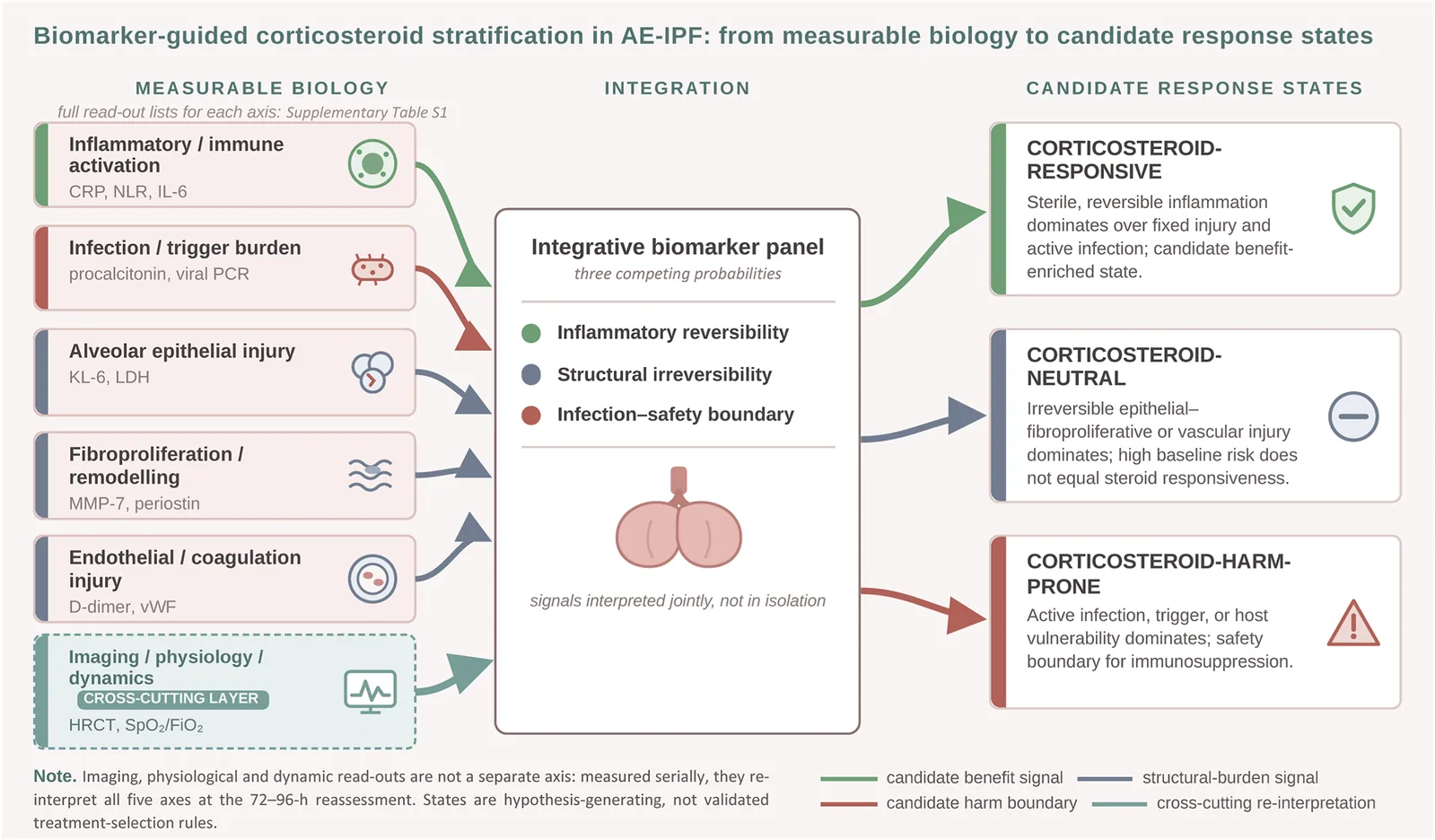
Biomarker-guided corticosteroid stratification in acute exacerbation of idiopathic pulmonary fibrosis. The schematic separates five primary biological axes—inflammatory and immune activation, infection or trigger burden, alveolar epithelial injury, fibroproliferation and remodelling, and endothelial and coagulation injury—from a cross-cutting layer of imaging, physiological, and dynamic read-outs. Only representative read-outs are shown; detailed lists are provided in Supplementary Table S1. Signals are interpreted jointly within an integrative panel across three domains: inflammatory reversibility, structural irreversibility, and the infection–safety boundary. Imaging, physiological, and dynamic read-outs do not constitute a separate biological axis; when measured serially, they refine interpretation across all five axes and may update the candidate response-state orientation at the 72–96-h reassessment. Integrated patterns nominate a provisional dominant orientation towards corticosteroid-responsive, corticosteroid-neutral, or corticosteroid-harm-prone biology, while allowing overlap and temporal transition between states. Green, slate-blue, red, and teal connectors represent candidate benefit signals, structural-burden signals, candidate harm boundaries, and cross-cutting reinterpretation, respectively. These states are hypothesis-generating orientations rather than validated treatment-selection rules.

**FIGURE 3:**
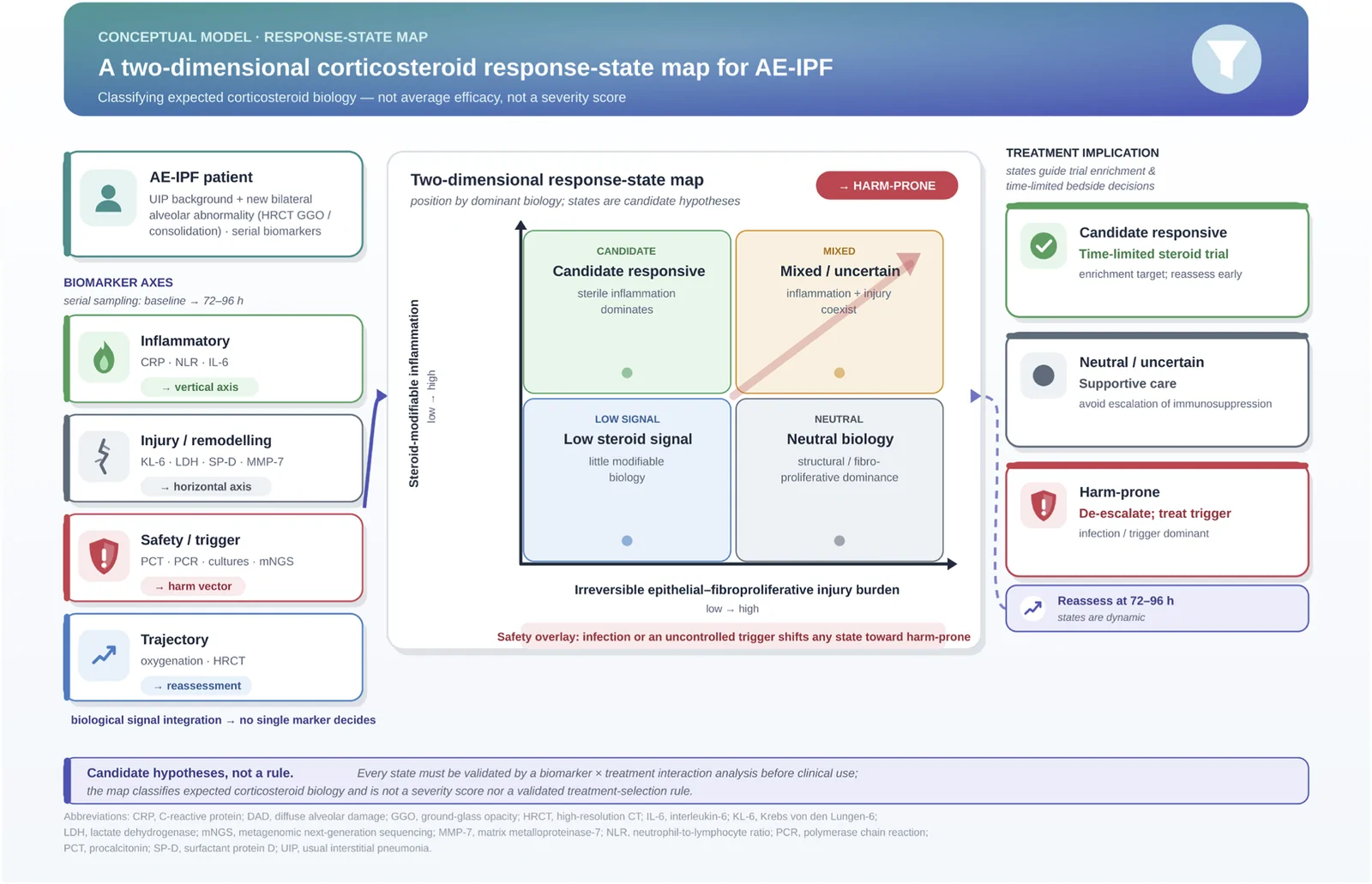
Two-dimensional corticosteroid response-state map for acute exacerbation of idiopathic pulmonary fibrosis. Each acute exacerbation event is positioned on a two-dimensional plane rather than assigned to a rigid diagnostic category. The vertical axis represents the degree of potentially corticosteroid-modifiable inflammation, ranging from low to high, whereas the horizontal axis represents the burden of irreversible epithelial and fibroproliferative injury, also ranging from low to high. The visual subregions correspond to the three higher-order candidate response states described in Section 5.2 and should not be interpreted as additional validated clinical categories. The candidate-responsive region corresponds to the corticosteroid-responsive state. The low-steroid-signal and neutral-biology regions represent subregions of the broader corticosteroid-neutral state. The mixed or uncertain region represents an indeterminate zone in which inflammatory and structural-injury signals coexist and for which early reassessment or trial stratification may be appropriate. Active infection, uncontrolled trigger burden, or major host vulnerability functions as a safety overlay that can shift any position on the plane toward the corticosteroid-harm-prone state.

These states should be interpreted as dominant biological orientations rather than mutually exclusive categories. They translate the biomarker axes developed in Section 4 into three provisional corticosteroid response states: corticosteroid-responsive, corticosteroid-neutral, and corticosteroid-harm-prone (Figure 2). The two-dimensional map in Figure 3 is intended to visualise these three higher-order states rather than introduce additional clinical categories. In that map, candidate-responsive corresponds to the corticosteroid-responsive state; low steroid signal and neutral biology represent subregions of the broader corticosteroid-neutral state; and mixed/uncertain denotes an indeterminate zone in which inflammatory and structural-injury signals coexist and should therefore be treated as a trial-enrichment or reassessment category rather than as a separate validated response state. The harm-prone state is represented as a safety overlay, because active infection, uncontrolled trigger burden, or major host vulnerability can shift any position on the plane toward net harm.

A corticosteroid-responsive state is hypothesised when an acute, predominantly sterile inflammatory process appears to dominate over fixed structural injury and active infection. Candidate features include abrupt clinical deterioration, high CRP or NLR, leucocytosis or neutrophilia interpreted in context, elevated ferritin, IL-6, IL-8, S100A8/A9, HMGB1, suPAR, sICAM-1, MIF, or IL-1β where available, and new GGO-predominant opacity on HRCT rather than dense consolidation. This state is strengthened by limited background fibrotic reserve loss, no convincing microbiological or aspiration driver, and physiological features suggesting potentially reversible injury rather than end-stage respiratory failure. Early improvement in SpO_2_/FiO_2_ or PaO_2_/FiO_2_, falling oxygen requirement, declining CRP, NLR, or LDH, and lack of radiological progression should be interpreted as post-initiation dynamic response markers rather than baseline predictors. This state is therefore an enrichment target for biomarker-stratified trials, not a validated indication for corticosteroids.

A corticosteroid-neutral state is hypothesised when the measurable biology points mainly to irreversible injury, limited reversibility, or loss of fibrotic reserve. This includes high epithelial-injury markers such as KL-6, SP-A, SP-D, GDF-15, sRAGE, HE4/WFDC2, or LDH; fibroproliferative and matrix-remodelling markers such as MMP-7, CCL18, osteopontin, periostin, or circulating fibrocyte-related signals; and endothelial or coagulation markers such as thrombomodulin, D-dimer, von Willebrand factor, angiopoietins, reduced protein C activity, or plasminogen activator inhibitor-1. Radiological features supporting this state include diffuse or dense acute involvement, a low GGO-to-consolidation ratio, extensive acute opacity burden, large background fibrosis volume, honeycombing, traction bronchiectasis, or quantitative radiomic evidence of advanced fibrotic or vascular injury. Severe baseline oxygenation failure, high oxygen or ventilatory requirement, poor ROX or oxygenation indices, and absent early improvement further support neutrality. The clinical importance of this state is to distinguish high-risk patients from plausibly steroid-responsive patients and to identify those in whom prolonged high-dose exposure is most likely to add toxicity without altering the dominant trajectory. The distinction between corticosteroid-neutral and corticosteroid-harm-prone states is therefore conceptual rather than a licence for prolonged exposure. A neutral state indicates low expected biological benefit because irreversible epithelial–fibroproliferative, vascular, or physiological injury predominates; a harm-prone state indicates that corticosteroids may actively worsen the dominant process, such as uncontrolled infection, aspiration-related injury, sepsis physiology, or marked host vulnerability. In practice, however, these states converge on a similar safety implication: neither should justify escalation of immunosuppression, and persistent neutrality during early reassessment should lower the threshold for tapering or discontinuation. Thus, neutrality may evolve into net harm if high-dose corticosteroids are continued without evidence of biological or physiological response.

A corticosteroid-harm-prone state is defined by evidence that immunosuppression may amplify the active driver of deterioration or the patient’s vulnerability to complications. Candidate features include elevated procalcitonin or presepsin, positive bacterial, viral, or fungal testing, bronchoalveolar lavage or mNGS findings adjudicated as clinically relevant rather than colonisation or contamination, aspiration signals such as pepsin or bile acids where available, drug toxicity, recent procedural triggers, sepsis physiology, recent or chronic immunosuppression, uncontrolled diabetes, severe frailty, multi-organ dysfunction, or other host factors that increase the hazards of corticosteroid exposure (Ding et al., 2013; Pitre et al., 2024; Zhan et al., 2024). In this state, inflammatory markers must be interpreted cautiously, because fever, neutrophilia, raised CRP, and new opacity may reflect infection-triggered injury rather than corticosteroid-modifiable sterile inflammation.

The temporal relationship between the neutral and harm-prone states deserves explicit statement, because the two are easily conflated. The harm-prone state is defined by intrinsic features present at the moment of assessment, namely, dominant infection, an uncontrolled trigger, or major host vulnerability, under which corticosteroids are expected to worsen the active disease process itself. The neutral state carries the opposite property: the dominant biology is unlikely to be modified by corticosteroids, so expected benefit is low, but corticosteroids do not accelerate the underlying process. The harm associated with a neutral state is therefore not intrinsic but treatment-emergent, accruing from cumulative exposure, including secondary infection, hyperglycaemia, myopathy, and delayed recognition of alternative diagnoses, when high-dose treatment is continued without response. A neutral state thus does not biologically become a harm-prone state; rather, prolonged ineffective exposure shifts its net risk–benefit balance towards harm, which is precisely the transition the 72–96-h reassessment is designed to detect (Table 1).

**TABLE 1:** Operational characteristics of the three candidate corticosteroid response states at baseline and at the 72–96-h reassessment.

Candidate state	Baseline features before corticosteroid exposure (biology and safety context)	Findings at 72–96 h reassessment	Expected corticosteroid effect and implication
Corticosteroid-responsive	Abrupt deterioration; high CRP or NLR; elevated IL-6, IL-8, S100A8/A9 or ferritin; new GGO-predominant HRCT abnormality; no convincing infection or trigger; limited background fibrosis	Improving SpO_2_/FiO_2_ or PaO_2_/FiO_2_; falling oxygen requirement; declining CRP, NLR or LDH; no radiological progression	Plausible benefit; supports a time-limited course with planned taper
Corticosteroid-neutral	Dominant epithelial, fibroproliferative or endothelial injury signals (KL-6, SP-D, GDF-15, LDH, MMP-7); dense or diffuse consolidation; extensive background fibrosis; severe baseline oxygenation failure	Absent early improvement; persistent or rising injury markers	Low expected benefit; no dominant excess-harm signal at baseline, but net harm may emerge if ineffective exposure is continued; taper, avoid escalation, and reconsider competing diagnoses
Corticosteroid-harm-prone	Elevated procalcitonin or presepsin; adjudicated bacterial, viral or fungal infection; aspiration markers; sepsis physiology; multi-organ dysfunction; major host vulnerability	Persistence or progression of the baseline harm driver, or treatment-emergent secondary infection or toxicity; deterioration during immunosuppression	Potential net harm from the outset or after a treatment-emergent transition; narrow or veto immunosuppression, treat the driver, and prioritise supportive care

States are hypothesis-generating orientations, not validated predictive or mutually exclusive categories. A neutral state denotes low expected benefit without a dominant baseline harm signal, whereas a harm-prone state denotes either a baseline safety threat or treatment-emergent harm during continued exposure; transitions may therefore occur between baseline and reassessment.

These response states are not treatment rules. They are structured hypotheses for separating probable benefit, neutrality, and harm, and they require prospective validation before clinical implementation.

### 5.3 Operational logic: two gates and one dynamic loop

The first gate is biological validity. The UIP/IPF background, acute time course, and new bilateral ground-glass opacity or consolidation must be confirmed, while competing or contributory diagnoses such as cardiac failure, fluid overload, pulmonary embolism, aspiration, and drug toxicity are actively assessed. This step is not merely diagnostic exclusion; it is essential for preserving biological validity and limiting phenotypic contamination that would obscure treatment effects in both practice and research.

The second gate is the safety boundary of immunosuppression. Procalcitonin, viral PCR, bacterial and fungal cultures, and, where feasible, bronchoalveolar sampling, mNGS, or aspiration markers should be interpreted before corticosteroids become an open-ended intervention. A positive test does not automatically prove causality, and a negative test does not prove sterility; the operative question is whether infection or another trigger is the dominant active driver at the time corticosteroid exposure is being continued.

The dynamic loop is reassessment at 72–96 h. This interval should be regarded as a pragmatic reassessment window rather than a validated biological threshold. Its rationale is that AE-IPF decisions are made under two simultaneous uncertainties: baseline biomarkers remain largely prognostic rather than predictive of corticosteroid response, and the optimal corticosteroid regimen itself is undefined. Recent data showing no adjusted mortality advantage of pulse over non-pulse corticosteroid regimens further suggest that dose escalation alone cannot substitute for early assessment of treatment trajectory (Hyung et al., 2024; Sasada et al., 2025).

The 72–96 h window is therefore best understood as a bedside test of trajectory. Improvement in SpO_2_/FiO_2_ or PaO_2_/FiO_2_, falling oxygen requirement, declining CRP, NLR, or LDH, and absence of radiological progression may support continuation of a time-limited corticosteroid course with planned taper. Conversely, failure to improve should trigger renewed search for occult infection, pulmonary embolism, fluid overload, aspiration, drug toxicity, or extensive non-reversible DAD, and should lower the threshold for early tapering or avoidance of further immunosuppressive escalation (Anan et al., 2022). This logic is supported by recent AE-IPF data showing that early SpO_2_/FiO_2_ improvement during Days 1–4 after steroid pulse therapy identifies patients with substantially lower short-term mortality (Sasada et al., 2025).

**FIGURE 4:**
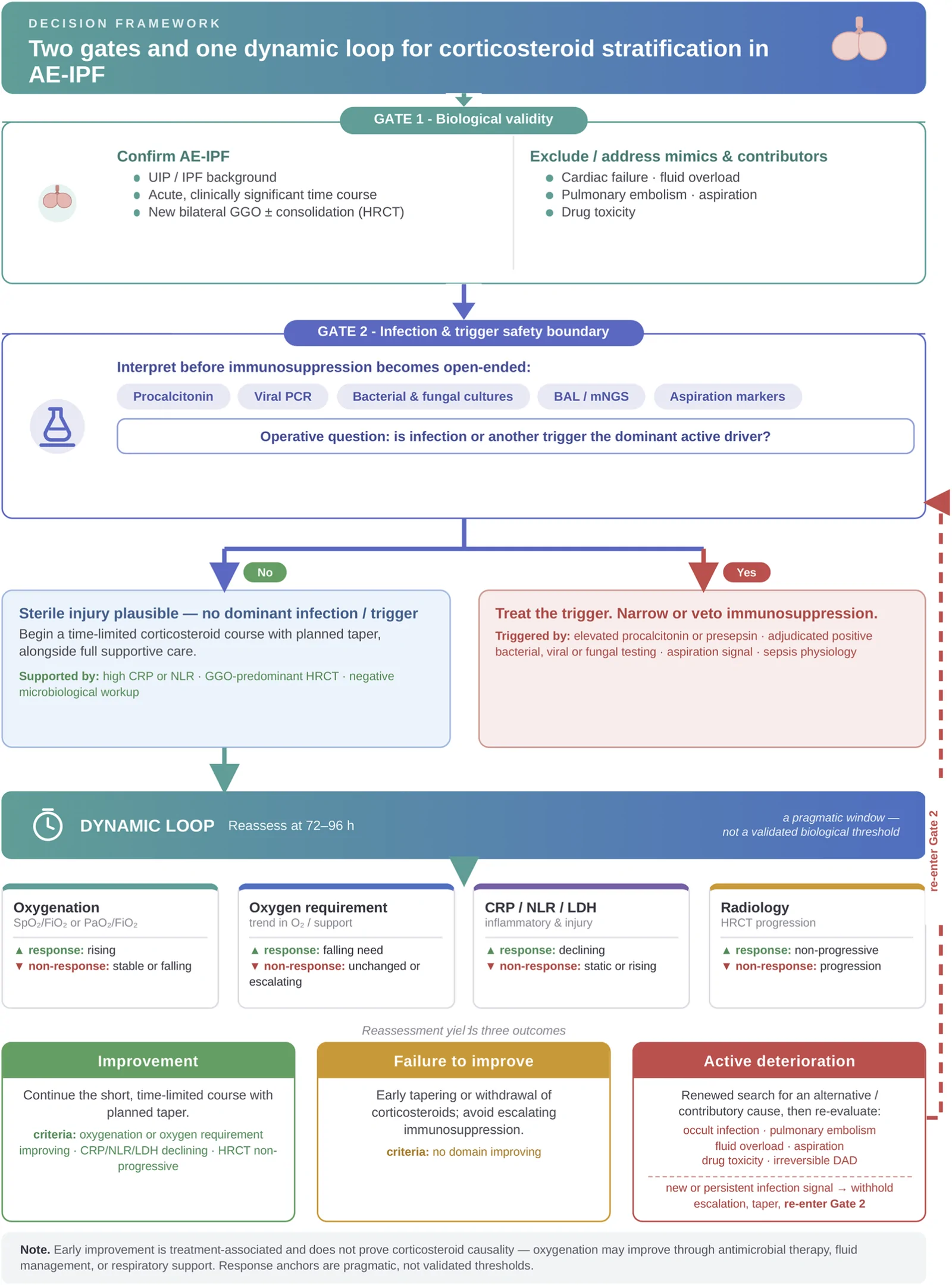
Investigational two-gate and 72–96-h dynamic reassessment framework for corticosteroid stratification in acute exacerbation of idiopathic pulmonary fibrosis. Gate 1 confirms clinical and radiological compatibility with acute exacerbation of idiopathic pulmonary fibrosis while assessing mimics and contributors such as cardiac failure, fluid overload, pulmonary embolism, aspiration, and drug toxicity. Gate 2 evaluates infection or another active trigger as a safety boundary for immunosuppression using microbiological, virological, biomarker, and clinical data. The 72–96-h loop represents a pragmatic reassessment window rather than a validated biological threshold. Serial oxygenation, oxygen requirement, C-reactive protein, neutrophil-to-lymphocyte ratio, lactate dehydrogenase, and radiological trajectory may define candidate continuation, tapering, or discontinuation rules for future prospective testing. Early improvement should be interpreted as treatment-associated rather than corticosteroid-causal, because trajectories may also reflect antimicrobial therapy, fluid management, respiratory support, or spontaneous fluctuation.

This response-adaptive step is intentionally asymmetric. Early improvement should not be interpreted as proof of corticosteroid causality, because oxygenation and inflammatory markers may improve through antimicrobial therapy, fluid management, ventilatory support, spontaneous fluctuation, or regression to the mean. Conversely, absence of improvement does not prove corticosteroid inefficacy in a strict causal sense, but it weakens the justification for continued high-dose immunosuppression when the expected benefit is uncertain and toxicity is cumulative. The purpose of the 72–96-h loop is therefore not to establish corticosteroid efficacy at the bedside, but to prevent corticosteroids from becoming an indefinite empirical default. It provides a pre-specified safety and futility checkpoint at which occult infection, alternative triggers, irreversible DAD, or structural-dominant biology should be reconsidered, and at which tapering or avoidance of further immunosuppressive escalation becomes more defensible. The full two-gate and 72–96-h dynamic reassessment framework is summarised in Figure 4.

The candidate response states can also be represented on a two-dimensional benefit–harm plane, with steroid-modifiable inflammation and irreversible epithelial–fibroproliferative injury as orthogonal axes and active infection or an uncontrolled trigger shifting any position towards a harm-prone state, as shown in Figure 3.

### 5.4 Validation roadmap

The framework will be useful only if it is made falsifiable. Prospective AE-IPF cohorts should record corticosteroid dose, timing, pulse use, tapering, antimicrobial exposure, antifibrotic background therapy, respiratory support, and goals-of-care decisions, together with serial biomarkers at presentation, 72–96 h, and day 7. HRCT should be visually scored or quantitatively segmented, and microbiological testing should distinguish detection, colonisation, and clinically adjudicated infection. The primary analytic target should be heterogeneity of treatment effect, tested as biomarker-by-treatment interaction, not another biomarker-by-mortality association (Hernán and Robins, 2016; Graham Linck et al., 2024).

A practical research sequence would be to derive candidate states using harmonised clinical, imaging, and multianalyte data; validate their transportability across centres; test whether corticosteroid effects differ across states; and only then embed a decision rule in biomarker-enriched or adaptive trials. This is consistent with current precision-medicine agendas in sepsis and ARDS, which emphasise richly phenotyped networks, real-time classifiers, adaptive designs, and learning platform trials (Shah et al., 2021; Leonard and Sinha, 2024; Practical, Panther, Traits, Incept, And Remap-Cap Investigators, 2024). Until such evidence exists, the proposed framework should guide trial design and cautious, time-limited clinical reasoning rather than function as a treatment protocol.

## 6 Discussion: challenges, controversies, and methodological limitations

### 6.1 The central limitation is inferential rather than descriptive

The major limitation of biomarker-guided corticosteroid stratification in AE-IPF is not the absence of candidate biomarkers, but the absence of evidence that any biomarker modifies the effect of corticosteroids. Most available studies describe associations between markers and mortality, future exacerbation, radiological severity, or short-term clinical trajectory. These associations are clinically useful, but they remain prognostic unless the corticosteroid effect differs across biomarker-defined strata. This distinction is not semantic. A marker that identifies a patient likely to die may still identify a patient unlikely to benefit from immunosuppression; conversely, a lower-risk patient with a steroid-modifiable inflammatory signal may be more responsive than a patient with extensive epithelial-fibroproliferative injury. The framework proposed here therefore rests on an unproven but testable premise: that AE-IPF contains heterogeneity of treatment effect, and that this heterogeneity can be captured by combinations of inflammatory, epithelial, infectious, radiological, and dynamic-response markers (Kent et al., 2018).

### 6.2 Confounding, indication bias, and time-dependent bias

The corticosteroid literature in AE-IPF is particularly vulnerable to confounding by indication. Sicker patients are more likely to receive high-dose corticosteroids, pulse therapy, rescue immunosuppression, intensive care, and mechanical ventilation; they are also more likely to die. Conventional adjustment can reduce but not eliminate this bias, especially when treatment timing, dose escalation, tapering, infection status, and goals-of-care decisions are incompletely captured. Dynamic-response markers introduce a second problem: immortal time bias. A patient must survive long enough to be classified as an early responder, to undergo tapering, or to have day-3/day-7 biomarkers measured. If this time structure is ignored, apparent benefits of response or tapering may partly reflect survival to the assessment point rather than a causal effect. Future observational studies should therefore be designed as target-trial emulations, explicitly defining time zero, eligibility, treatment strategies, grace periods, follow-up, intercurrent events, and analytic handling of time-varying treatment and biomarkers (Hernán et al., 2016).

### 6.3 Heterogeneity of definition, population, and corticosteroid exposure

AE-IPF is a broadened clinical construct rather than a single biological entity. The 2016 definition intentionally includes both idiopathic and triggered events, increasing clinical realism but also increasing biological heterogeneity (Collard et al., 2016). Studies differ in whether they include infection-triggered exacerbations, post-procedural events, acute exacerbations of non-IPF fibrotic ILD, patients receiving antifibrotics, ICU-level respiratory failure, or patients in whom invasive ventilation is withheld. Corticosteroid exposure is equally heterogeneous: dose, pulse use, timing from symptom onset, concomitant antibiotics, additional immunosuppressants, and tapering schedules vary widely. These differences make pooled average effects difficult to interpret. These differences also limit the transportability of biomarker thresholds and treatment effects across regions and healthcare systems (Dahabreh et al., 2021). Background antifibrotic therapy is a particularly consequential source of such heterogeneity, because it may modify disease biology, biomarker levels, and corticosteroid responsiveness simultaneously; it is therefore considered separately in Section 6.4.

### 6.4 Antifibrotic background therapy: confounder, stratification variable, or effect modifier?

Background antifibrotic therapy intersects disease biology, biomarker interpretation, and treatment response, and warrants dedicated discussion. Many patients experiencing AE-IPF in contemporary practice are already receiving nintedanib or pirfenidone, yet management during the exacerbation is variable and non-random: survey respondents differed on continuing, switching, or discontinuing these agents (Kreuter et al., 2020); some real-world cohorts continued antifibrotics in nearly all patients (Isshiki et al., 2021); and a propensity-matched nationwide database study found no short-term outcome advantage of continuing nintedanib over discontinuation (Shuto et al., 2026), although such comparisons are themselves confounded by indication.

Antifibrotic therapy may also reshape the biology the framework is trying to measure. Nintedanib reduces the risk of adjudicated exacerbations (Kreuter et al., 2019), so an event occurring despite treatment may represent a breakthrough state selected for treatment-refractory biology, plausibly distinct from an exacerbation in an antifibrotic-naïve patient. Because both agents act beyond matrix deposition, on growth-factor receptor signalling, macrophage function, and cytokine production, they could blunt the inflammatory and endothelial signals nominating the corticosteroid-responsive state, shifting treated patients towards corticosteroid-neutral biology; this remains untested.

Antifibrotic exposure may also alter the biomarkers themselves. Serial studies show that KL-6, SP-D, MMP-7, CCL18, and periostin behave differently under antifibrotic treatment than in untreated cohorts (Majewski et al., 2021), and, as Sections 4.2 and 4.3 emphasise, the few markers from these axes examined for treatment-effect modification were tested against antifibrotics rather than corticosteroids. A threshold derived in an antifibrotic-naïve cohort cannot be assumed to carry the same meaning in a treated one; biomarker distributions should therefore be reported stratified by antifibrotic exposure, and background status specified as part of the measurement context in context-of-use validation.

We therefore propose that background antifibrotic therapy be handled at three distinct levels. As a confounder, it should be recorded (agent, dose, duration, and continuation or interruption during the exacerbation) and adjusted for in any biomarker-by-treatment interaction analysis, because exposure tracks with disease duration, care setting, and treatment culture. As a stratification variable, it should be incorporated into randomisation in corticosteroid trials so that allocation is balanced within antifibrotic-defined strata. As a candidate effect modifier, it should be examined through pre-specified antifibrotic-stratified interaction analyses, while recognising the sample-size burden of three-way interactions. We deliberately do not elevate antifibrotic background to a fourth decision domain, because there is no evidence that it identifies corticosteroid-responsive, corticosteroid-neutral, or corticosteroid-harm-prone biology; it is a source of structured heterogeneity to be measured and modelled, not yet a selection criterion.

### 6.5 Generalisability and transportability of the evidence

A substantial proportion of the biomarker evidence synthesised here originates from Japanese cohorts, reflecting both the structure of the AE-IPF literature and the major contribution of Japanese investigators. This geographic concentration may limit transportability. Frequencies of some IPF-associated variants, particularly MUC5B rs35705950, differ across ancestry groups, while biomarker distributions and clinically useful thresholds may also vary. Although KL-6 has been evaluated outside Japan, much of its AE-IPF-specific evidence and routine clinical adoption remains concentrated in Japan and selected healthcare systems. Regional differences in assay availability, referral pathways, and treatment practices may further affect biomarker performance. Thresholds, effect estimates, and the contribution of individual axes should therefore not be assumed to transfer unchanged across populations. Before clinical implementation, the framework requires external validation in geographically and ethnically diverse cohorts, ideally through multinational prospective registries, with assessment of calibration and local recalibration where necessary.

### 6.6 Biomarker timing, compartment, assay portability, and model overfitting

A stratification framework is only as portable as the measurements on which it relies. The same biomarker may carry different information at stable baseline, exacerbation onset, pre-treatment, 72–96 h, or day 7. Compartment is also decisive: serum, plasma, bronchoalveolar lavage, tissue, HRCT-derived features, and oxygenation trajectories are not interchangeable. Assay standardisation remains uneven, particularly for markers such as KL-6, SP-D, periostin, cytokines, and multiplex inflammatory panels. A cut-off derived on one platform or in one healthcare system cannot be assumed to transfer internationally. The appropriate standard is therefore not biomarker discovery alone, but context-of-use validation: what decision the marker is meant to support, at what time point, with which assay, in which population, and against what clinical alternative. The same caution applies to radiomics and machine-learning classifiers. These tools are attractive because they can integrate high-dimensional imaging and clinical data, but without external validation, calibration, reporting transparency, and assessment of clinical utility, they risk becoming sophisticated prognostic scores rather than deployable treatment-selection instruments (Collins et al., 2024).

### 6.7 The infection-inflammation boundary is the hardest safety problem

The most consequential controversy is the boundary between sterile inflammation and infection-triggered injury. The framework’s benefit axis and harm axis collide precisely here. Fever, neutrophilia, elevated CRP, and raised NLR may suggest a steroid-responsive inflammatory phenotype, but the same findings may reflect bacterial, viral, fungal, or aspiration-related injury. Molecular diagnostics do not fully solve the problem: mNGS and PCR increase detection but cannot by themselves distinguish pathogen, colonisation, contamination, or bystander microbiome disruption (Gu et al., 2019; Zhan et al., 2024). Thus, the clinically necessary concept is dominance: whether infection or sterile inflammation is the principal active driver at the moment corticosteroids are being continued or escalated. At present, dominance is adjudicated clinically rather than measured biologically. This uncertainty argues for a conservatively weighted safety boundary: infection signals should not merely be covariates in a benefit model, but potential veto signals against ongoing immunosuppression. A key methodological task for future studies is therefore to convert clinical adjudication of “dominance” into operational biomarker rules. Such rules should be pre-specified before analysis and should combine three components: first, objective trigger evidence, including procalcitonin or presepsin, viral PCR, bacterial or fungal culture, BAL or mNGS results, aspiration markers, and sepsis physiology; second, inflammatory-benefit evidence, including CRP or NLR dynamics, cytokine profiles where available, GGO-predominant HRCT abnormality, and early oxygenation trajectory; and third, structural-irreversibility evidence, including KL-6, LDH, SP-D, MMP-7, background fibrosis burden, dense consolidation, and poor physiological reserve. In target-trial emulations or prospective cohorts, these elements could be translated into explicit rules, such as an infection-dominant veto rule, an inflammatory-enrichment rule, and a non-response futility rule at 72–96 h. Importantly, these rules should be derived, calibrated, and externally validated against biomarker-by-treatment interaction, rather than assumed to be correct from expert judgement alone.

### 6.8 Practical implementation: availability, turnaround time, and workflow integration

Implementation depends on local availability, turnaround time, and cost, which do not always align. A core tier available in most centres managing AE-IPF comprises oxygenation indices, blood cell counts and NLR, CRP, LDH, ferritin, D-dimer, procalcitonin where available, HRCT, routine microbiology, and rapid respiratory PCR. Most non-culture results are available within hours and can support both baseline assessment and the 72–96-h reassessment. A limited-availability tier includes KL-6, presepsin, and cytokine or multiplex inflammatory panels. KL-6 and presepsin can be rapid on dedicated in-house platforms but remain unavailable or require send-out testing in many systems, whereas multiplex panels are commonly batch-run. A specialist or research tier includes mNGS, bronchoalveolar pepsin or bile acids, S100A8/A9, suPAR, GDF-15, and MMP-7. mNGS may return within 24–72 h in specialised centres, although external workflows can take longer; BAL-dependent tests are additionally constrained by procedural feasibility in severe hypoxaemia. Any assay intended to influence day-3 or day-4 decisions should therefore be ordered at presentation and locally verified to return before that decision point. Rapid respiratory PCR, point-of-care CRP or procalcitonin where available, and serial bedside SpO_2_/FiO_2_ can support the time-critical safety gate. Section 5 reassessment rules could also be incorporated into electronic health records as a day-3 prompt integrating oxygenation trajectory, CRP or NLR change, and pending microbiology. Such decision-support tools require prospective validation and should accommodate missing data, local assay availability, and alert fatigue while supporting, rather than automating, corticosteroid decisions.

## 7 Future directions: from prognostic association to treatment-selecting evidence

### 7.1 Use randomised evidence to ask the predictive question

The immediate priority is to exploit randomisation not only to estimate the average corticosteroid effect, but to test whether that effect differs across biologically defined strata. The ongoing EXAFIP2 trial (NCT05674994), a placebo-controlled trial of glucocorticoids in AE-IPF, is therefore important not simply because it may clarify whether corticosteroids work on average, but because it creates the cleanest setting in which biomarker-by-treatment interaction can be examined without confounding by indication (Naccache et al., 2022; Fondation Hôpital Saint-Joseph, 2026). Its interpretability, however, will depend on whether baseline inflammatory, epithelial-injury, infection, radiological, and early physiological markers are captured with sufficient granularity. If biomarker analyses are treated only as exploratory associations with mortality, the trial risks answering the old question. If interaction is pre-specified, it can begin to answer the clinically decisive one: in whom does corticosteroid exposure alter outcome?

### 7.2 Emulate treatment strategies, not treatment labels

Observational data will remain necessary because AE-IPF is uncommon, rapidly fatal, and difficult to randomise at scale. Yet future observational studies should emulate explicit target trials rather than compare ill-defined “steroid” and “non-steroid” groups. Target-trial emulation refers to the explicit specification of a hypothetical randomized trial protocol—including time zero, eligibility criteria, treatment strategies, follow-up, outcomes, and analytic approach—and then applying that protocol as closely as possible to observational data. The strategies of interest are clinically concrete: pulse *versus* non-pulse regimens, early *versus* delayed initiation, short planned taper *versus* prolonged high-dose exposure, and response-adaptive continuation *versus* early discontinuation. Each emulation should define time zero, eligibility, grace periods, intercurrent events, and dynamic biomarkers before analysis, using time-varying methods where treatment and response evolve together (Hernán et al., 2016). This would convert existing real-world data from a source of confounded associations into a bridge between current practice and future trials.

### 7.3 Build a prospective AE-IPF precision cohort

A multicentre prospective registry is the necessary infrastructure between hypothesis and trial. It should be deliberately lean but biologically complete: harmonised AE-IPF definitions; adjudication of triggered *versus* idiopathic events; standardised recording of corticosteroid dose, pulse use, tapering, antimicrobials, antifibrotic therapy, respiratory support, and limitations of care; and serial sampling at presentation, pre-treatment where possible, 72–96 h, day 7, and day 14. Biomarkers should be anchored to context of use, not merely collected: inflammatory markers to estimate steroid-modifiable activity, epithelial and remodelling markers to estimate irreversible injury, microbiological markers to define the harm boundary, and quantitative HRCT or radiomics to measure acute opacity and background fibrotic burden. Such a cohort would allow the proposed response states to be empirically derived, externally validated, and tested for transportability across regions, assay platforms, and treatment cultures (Dahabreh et al., 2021; Collins et al., 2024). Future prospective cohorts should capture patient-centred outcomes alongside biological and clinical endpoints. Health-related quality of life and symptom burden could be assessed using validated instruments such as the King’s Brief Interstitial Lung Disease questionnaire or the IPF-specific St George’s Respiratory Questionnaire, supplemented by validated dyspnoea and cough measures. Functional recovery could be assessed using activities-of-daily-living measures and, once clinically feasible, the 6-min walk distance. Although these outcomes are unlikely to guide the initial acute corticosteroid decision, serial assessment after clinical stabilisation, at discharge, and during follow-up could characterise recovery among survivors and capture outcomes that matter directly to patients. Future evaluations should therefore determine not only whether biomarker-guided stratification affects mortality or biological response states, but also whether it improves symptoms, functional status, and health-related quality of life.

### 7.4 Convert composite signatures into clinical decision rules

The field should move from single biomarkers to composite, interpretable signatures. A useful model would not simply predict death; it would estimate the relative probabilities of corticosteroid-responsive inflammation, corticosteroid-neutral structural injury, and corticosteroid-related harm. Quantitative imaging, oxygenation trajectory, CRP/NLR dynamics, epithelial injury markers, and infection adjudication should therefore be integrated into parsimonious classifiers that can be used at the bedside and audited across settings. Model development must include external validation, calibration, transparent reporting, and assessment of clinical utility, including decision-curve analysis, because a statistically accurate model may still be clinically harmful if it increases unnecessary immunosuppression or misses infection-dominant events (Vickers and Elkin, 2006).

### 7.5 Test the strategy in biomarker-enriched and adaptive trials

The mature test of the framework is not another unselected corticosteroid trial, but a biomarker-enriched or adaptive trial in which the stratification logic is part of the design. A biomarker-enriched design could randomise patients with a dominant inflammatory, non-infectious phenotype, thereby testing corticosteroids where benefit is most biologically plausible. A platform or master-protocol design could evaluate multiple corticosteroid strategies and allow early stopping for futility or harm within predefined biological strata (Woodcock and LaVange, 2017; Angus et al., 2020). Most importantly, the 72–96 h reassessment proposed in Section 5 should be tested as a treatment strategy: continuation, tapering, or discontinuation governed by early oxygenation, inflammatory, radiological, and safety trajectories. This requires careful estimand definition, because death, mechanical ventilation, treatment escalation, transplant evaluation, and withdrawal of life-sustaining treatment are not nuisances but clinically meaningful intercurrent events.

Taken together, these steps define a research programme rather than a wish list. Randomised trials provide the cleanest interaction tests; target-trial emulation extracts disciplined causal information from current practice; prospective registries derive and validate biological states; composite signatures turn biology into decisions; and adaptive trials test whether those decisions improve outcomes. The goal is not to prove that corticosteroids are uniformly good or bad in AE-IPF, but to replace indiscriminate exposure with treatment-selecting evidence. Although developed for corticosteroid decisions, the framework is not intrinsically treatment-specific. Its three decision domains—modifiable inflammation, irreversible epithelial–fibroproliferative injury, and the safety boundary—describe the biology that any acute or concurrent therapy must confront, and treatment-effect modification within these axes has precedent beyond corticosteroids: baseline SP-D identified the subgroup of patients in whom pirfenidone slowed functional decline, and higher baseline periostin predicted a more favourable response to nintedanib. As evidence accumulates for antifibrotic continuation during exacerbation, additional immunomodulatory treatments, and differentiated supportive-care strategies, the same axis structure could evolve into a broader treatment-stratification tool, with each therapy tested for biomarker-by-treatment interaction within a shared biological coordinate system.

## 8 Conclusion

AE-IPF remains one of the most lethal and therapeutically unresolved complications of IPF. Although systemic corticosteroids remain widely used, their evidentiary foundation is weak, their average effect in unselected patients is uncertain, and their potential for harm is increasingly difficult to ignore. The central problem is not simply that better biomarkers are needed, but that the current biomarker literature has largely generated prognostic rather than predictive evidence. Markers of epithelial injury, inflammation, infection, radiological extent, and physiological deterioration can identify risk, but they do not yet identify patients whose outcomes are modified by corticosteroids.

The most clinically useful way forward is therefore to reframe AE-IPF as a heterogeneous treatment-response syndrome rather than a uniform indication for empirical immunosuppression. A biomarker-guided framework should distinguish three questions that are often conflated: whether steroid-modifiable inflammation is present, whether irreversible epithelial-fibroproliferative injury predominates, and whether infection or host vulnerability makes immunosuppression unsafe. In this context, early dynamic reassessment is essential, because baseline markers alone are unlikely to resolve the corticosteroid decision. The goal is not to replace clinical judgement with a premature algorithm, but to make that judgement more biologically explicit, safer, and testable.

At present, the proposed stratification framework should be regarded as a disciplined hypothesis rather than a validated treatment rule. Its value lies in clarifying what evidence the field now needs: randomised and treatment-aware observational studies that record corticosteroid timing, dose, tapering, infection status, serial biomarkers, quantitative imaging, and early physiological trajectories; analyses designed to test biomarker-by-treatment interaction; and eventually biomarker-enriched or adaptive trials. Only such evidence can move AE-IPF care beyond the current binary debate over whether corticosteroids “work” on average, toward the more clinically meaningful question of which patients, if any, benefit from them, which are unlikely to benefit, and which may be harmed.

## Data Availability

The original contributions presented in the study are included in the article/Supplementary Material, further inquiries can be directed to the corresponding author.

## Glossary

AE-IPF: acute exacerbation of idiopathic pulmonary fibrosis
AI: artificial intelligence
ARDS: acute respiratory distress syndrome
ATS/ERS/JRS/ALAT: American Thoracic Society/European Respiratory Society/Japanese Respiratory Society/Latin American Thoracic Association
BAL: bronchoalveolar lavage
CCL18: C-C motif chemokine ligand 18
CRP: C-reactive protein
CT: computed tomography
DAD: diffuse alveolar damage
EXAFIP: cyclophosphamide-added-to-glucocorticoids trial in AE-IPF
EXAFIP2: placebo-controlled glucocorticoid trial in AE-IPF
**FiO**_**2**_: fraction of inspired oxygen
GDF-15: growth differentiation factor-15
GGO: ground-glass opacity
HE4: human epididymis protein 4
HMGB1: high-mobility group box 1
HR: hazard ratio
HRCT: high-resolution computed tomography
ICU: intensive care unit
IL: interleukin
ILD: interstitial lung disease
IPF: idiopathic pulmonary fibrosis
KL-6: Krebs von den Lungen-6
LDH: lactate dehydrogenase
MIF: macrophage migration inhibitory factor
MMP-7: matrix metalloproteinase-7
mNGS: metagenomic next-generation sequencing
NLR: neutrophil-to-lymphocyte ratio
PAI-1: plasminogen activator inhibitor-1
**PaO**_**2**_: arterial partial pressure of oxygen
PCR: polymerase chain reaction
PROFILE: Prospective Observation of Fibrosis in the Lung Clinical Endpoints
RAGE: receptor for advanced glycation end-products
RCT: randomised controlled trial
ROX: respiratory rate-oxygenation index
S100A8/A9: S100 calcium-binding proteins A8/A9
sICAM-1: soluble intercellular adhesion molecule-1
SP-A/SP-D: surfactant proteins A/D
**SpO**_**2**_: peripheral oxygen saturation
sRAGE: soluble receptor for advanced glycation end-products
suPAR: soluble urokinase plasminogen activator receptor
TGF-β: transforming growth factor beta
UIP: usual interstitial pneumonia
WFDC2: WAP four-disulfide core domain 2
