# Supplementary material for: A biomarker-guided framework for corticosteroid treatment stratification in acute exacerbation of idiopathic pulmonary fibrosis
Source: Front Pharmacol. 2026 Aug 24;17:1934548. doi: 10.3389/fphar.2026.1934548 (PMC13504195; doi:10.3389/fphar.2026.1934548)
Supplement: Supplementary file 1 [file Table1.docx]

Supplementary Material

# 1 Supplementary Figures and Tables

## 1.1 Supplementary Table S1. Candidate biological axes and biomarker roles for corticosteroid stratification in acute exacerbation of idiopathic pulmonary fibrosis.

| **Biological / response axis** | **Representative read-outs** | **Dominant current evidence role** | **Candidate corticosteroid-response implication** | **How to interpret within a panel** | **Key evidence gap** | **Refer-ences** |
| --- | --- | --- | --- | --- | --- | --- |
| **Alveolar epithelial injury** | KL-6; SP-A/SP-D; GDF-15; sRAGE; HE4/WFDC2; LDH | Mainly diagnostic, risk, and prognostic; reflects epithelial damage and barrier injury. | More likely corticosteroid-neutral than corticosteroid-responsive when strongly dominant. | High values suggest extensive acute structural injury or failed repair, especially with severe hypoxaemia and broad HRCT abnormality. | Needs biomarker-by-corticosteroid interaction testing to determine whether high injury signals predict no benefit. | (Tian et al., 2022; Balci et al., 2023; Wang et al., 2023, 2024; Ebert et al., 2024; Jenkins et al., 2024; Oldham et al., 2024; Tzang et al., 2025) |
| **Inflammatory and immune activation** | CRP; NLR; neutrophil count; IL-6; IL-8; S100A8/A9; ferritin; suPAR; macrophage-related mediators | Mainly prognostic; biologically plausible marker of steroid-modifiable activity. | Candidate benefit signal if inflammation appears sterile and reversible. | Interpret only together with infection markers, HRCT pattern, and early oxygenation trajectory; inflammation alone is not proof of responsiveness. | No validated inflammatory signature has yet shown differential corticosteroid effect in AE-IPF. | (Balci et al., 2023; Wang et al., 2023; Ebert et al., 2024; Oldham et al., 2024; Zheng et al., 2024) |
| **Infection and trigger burden** | Procalcitonin; presepsin; bacterial culture; viral PCR; fungal markers; BAL/mNGS; aspiration markers such as pepsin or bile acids; drug/procedure triggers | Safety or harm marker; helps adjudicate triggered events and competing biology. | Candidate corticosteroid-harm-prone state when infection or another active trigger dominates. | A positive signal should narrow or veto ongoing immunosuppression unless infection is clinically judged non-dominant or controlled. | Microbial detection does not prove causation; studies must distinguish infection, colonisation, contamination, and dysbiosis. | (Takeshita et al., 2024, 2025; Srivali and De Giacomi, 2025) |
| **Endothelial, vascular, and coagulation injury** | Thrombomodulin; D-dimer; von Willebrand factor; angiopoietins; protein C; PAI-1 | Primarily prognostic severity signal; reflects alveolar-capillary injury and coagulation activation. | Uncertain; not a standalone corticosteroid benefit marker. | May identify severe DAD-like injury and high baseline risk, but must be integrated with inflammatory and infection axes. | Unselected pathway-directed strategies have not established benefit; role in composite endotypes remains unvalidated. | (Takeshita et al., 2024; Pang et al., 2025) |
| **Fibroproliferation and remodelling** | MMP-7; periostin; osteopontin; CCL18; circulating fibrocytes; extracellular-matrix turnover markers | Risk and prognostic; reflects background fibrotic burden and disordered repair. | Candidate corticosteroid-neutral state when structural remodelling dominates. | High remodelling burden may dilute apparent steroid benefit in unselected cohorts, especially with limited inflammatory activity. | Requires testing whether remodelling-high / inflammation-low profiles identify patients unlikely to benefit. | (Balci et al., 2023; Wang et al., 2023; Ebert et al., 2024; Iturbe-Fernández et al., 2024; Jenkins et al., 2024; Maher et al., 2024; Oldham et al., 2024; Zheng et al., 2024; Li et al., 2025; Wu et al., 2025) |
| **Imaging, physiology, and dynamic response** | HRCT distribution and extent of new GGO/consolidation; GGO-to-consolidation balance; background fibrosis; PaO2/FiO2 or SpO2/FiO2; oxygen requirement; 72-96 h trajectory | Diagnostic and prognostic at baseline; dynamic-response marker after treatment begins. | Most useful for deciding continuation, tapering, or discontinuation rather than baseline eligibility alone. | Improving oxygenation, falling oxygen need, declining CRP/NLR/LDH, and non-progressive imaging support a time-limited course; non-response should trigger reassessment. | Early improvement is treatment-associated, not causal proof; response-adaptive rules need prospective validation. | (Takeshita et al., 2024, 2025; Pang et al., 2025; Zhang et al., 2025; Park et al., 2026) |

**Abbreviations:** AE-IPF, acute exacerbation of idiopathic pulmonary fibrosis; BAL, bronchoalveolar lavage; CRP, C-reactive protein; DAD, diffuse alveolar damage; GDF-15, growth differentiation factor-15; GGO, ground-glass opacity; HE4/WFDC2, human epididymis protein 4; HRCT, high-resolution computed tomography; KL-6, Krebs von den Lungen-6; LDH, lactate dehydrogenase; mNGS, metagenomic next-generation sequencing; MMP-7, matrix metalloproteinase-7; NLR, neutrophil-to-lymphocyte ratio; PAI-1, plasminogen activator inhibitor-1; PCR, polymerase chain reaction; sRAGE, soluble receptor for advanced glycation end-products; SP-A/SP-D, surfactant proteins A/D; suPAR, soluble urokinase plasminogen activator receptor.

**Note:** Current AE-IPF biomarkers are predominantly diagnostic, risk, prognostic, safety, or dynamic-response markers. None should be presented as a validated predictive biomarker for corticosteroid response unless a biomarker-by-treatment interaction has been demonstrated.

**Supplementary references**

Balci, A., Düz, M. E., Vurmaz, A., Çilekar, Ş., and Kaya, F. (2023). Comprehensive biomarker analysis of patients with idiopathic pulmonary fibrosis and interstitial lung disease with healthy individuals. *Eur. Rev. Med. Pharmacol. Sci.* 27, 5468–5479. doi: 10.26355/eurrev_202306_32783

Ebert, C., Walsh, A. M., Sereda, L., Wilson, C. L., Schafer, P. H., Fischer, A., et al. (2024). Circulating biomarker analyses in a longitudinal cohort of patients with IPF. *Am. J. Physiol., Lung Cell. Mol. Physiol.* 326, L303–L312. doi: 10.1152/ajplung.00222.2023

Iturbe-Fernández, D., Pulito-Cueto, V., Mora-Cuesta, V. M., Remuzgo-Martínez, S., Ferrer-Pargada, D. J., Genre, F., et al. (2024). Osteopontin as a biomarker in interstitial lung diseases. *Biomedicines* 12, 1108. doi: 10.3390/biomedicines12051108

Jenkins, R. G., Cottin, V., Nishioka, Y., Noth, I., White, E. S., Ittrich, C., et al. (2024). Effects of nintedanib on circulating biomarkers of idiopathic pulmonary fibrosis. *ERJ Open Res.* 10, 558–2023. doi: 10.1183/23120541.00558-2023

Li, R. M., Tan, D. B. A., Tedja, C., Cooper, W. A., Jo, H. E., Grainge, C., et al. (2025). Pre-treatment MMP7 predicts progressive idiopathic pulmonary fibrosis in antifibrotic treated patients. *Respirology* 30, 504–514. doi: 10.1111/resp.14894

Maher, T. M., Jenkins, R. G., Cottin, V., Nishioka, Y., Noth, I., Selman, M., et al. (2024). Circulating biomarkers and progression of idiopathic pulmonary fibrosis: Data from the INMARK trial. *ERJ Open Res.* 10, 335–2023. doi: 10.1183/23120541.00335-2023

Oldham, J. M., Huang, Y., Bose, S., Ma, S.-F., Kim, J. S., Schwab, A., et al. (2024). Proteomic biomarkers of survival in idiopathic pulmonary fibrosis. *Am. J. Respir. Crit. Care Med.* 209, 1111–1120. doi: 10.1164/rccm.202301-0117OC

Pang, X., Cheng, L., Huang, L., He, W., Liu, S., Wang, Y., et al. (2025). Radiomics derived from HRCT can predict mortality in patients with acute exacerbation of idiopathic pulmonary fibrosis. *J. Thorac. Dis.* 17, 4991–5001. doi: 10.21037/jtd-2025-194

Park, S., Kim, M.-J., Lee, J. H., Hwang, H. J., Lee, S. M., Chae, E. J., et al. (2026). Quantitative CT imaging in progressive pulmonary fibrosis: Clinical usefulness and meaningful threshold definition. *Chest* 169, 1255–1266. doi: 10.1016/j.chest.2025.11.031

Srivali, N., and De Giacomi, F. (2025). Procalcitonin in acute exacerbation of interstitial lung disease: A systematic review of current evidence. *Heart Lung: J. Crit. Care* 73, 221–227. doi: 10.1016/j.hrtlng.2025.05.014

Takeshita, Y., To, M., Kurosawa, Y., Furusho, N., Kinouchi, T., Tsushima, K., et al. (2024). Usefulness of combined measurement of surfactant protein D, thrombin-antithrombin III complex, D-dimer, and plasmin-α2 plasmin inhibitor complex in acute exacerbation of interstitial lung disease: A retrospective cohort study. *J. Clin. Med.* 13, 2427. doi: 10.3390/jcm13082427

Takeshita, Y., To, Y., To, M., Furusho, N., Kurosawa, Y., Kinouchi, T., et al. (2025). Potential utility of combined presepsin and LDH tracking for predicting therapeutic efficacy of steroid pulse therapy in acute exacerbation of interstitial lung diseases: A pilot study. *J. Clin. Med.* 14, 3068. doi: 10.3390/jcm14093068

Tian, M., Meng, K., Gao, Y., Zhang, J., Xie, M., Tian, Y., et al. (2022). Elevated serum human epididymis protein 4 is associated with disease severity and worse survival in idiopathic pulmonary fibrosis: A cohort study. *Ann. Transl. Med.* 10, 992. doi: 10.21037/atm-22-4042

Tzang, C.-C., Lin, W.-C., Huang, E. S., Kang, Y.-F., Cheng, Y.-H., Tzang, B.-S., et al. (2025). Interstitial lung disease biomarkers: A systematic review and meta-analysis. *Clin. Chim. Acta; Int. J. Clin. Chem.* 577, 120473. doi: 10.1016/j.cca.2025.120473

Wang, L., Zhu, M., Li, Y., Yan, P., Li, Z., Chen, X., et al. (2023). Serum proteomics identifies biomarkers associated with the pathogenesis of idiopathic pulmonary fibrosis. *Mol. Cell. Proteom.: MCP* 22, 100524. doi: 10.1016/j.mcpro.2023.100524

Wang, T., Yao, Y., Wang, Y., Wei, W., Yin, B., Huang, M., et al. (2024). Evaluating the diagnostic and therapeutic significance of KL-6 in patients with interstitial lung diseases. *Heliyon* 10, e27561. doi: 10.1016/j.heliyon.2024.e27561

Wu, S., Liu, H., Yin, S., Wang, Y., Liu, X., and Ding, Z. (2025). The significance of periostin in the diagnosis of idiopathic pulmonary fibrosis and prediction of acute exacerbations. *J. Thorac. Dis.* 17, 1364–1376. doi: 10.21037/jtd-24-1882

Zhang, T., Lee, J.-H., Kim, S. R., Chung, J. H., and Jin, G. Y. (2025). What role does artificial intelligence-driven quantitative analysis of chest computed tomography play in providing pulmonary function for idiopathic pulmonary fibrosis patients undergoing pirfenidone treatment? *Quant. Imaging Med. Surg.* 15, 6604–6615. doi: 10.21037/qims-2025-380

Zheng, Z., Peng, F., and Zhou, Y. (2024). Biomarkers in idiopathic pulmonary fibrosis: Current insight and future direction. *Chin. Med. J. Pulm. Crit. Care Med.* 2, 72–79. doi: 10.1016/j.pccm.2024.04.003
